# Structural variation affecting DNA backbone interactions underlies adaptation of B3 DNA binding domains to constraints imposed by protein architecture

**DOI:** 10.1093/nar/gkab257

**Published:** 2021-04-19

**Authors:** Haiyan Jia, Masaharu Suzuki, Donald R McCarty

**Affiliations:** Horticultural Sciences Department, Plant Molecular and Cellular Biology Program, University of Florida, Gainesville, FL 32611-0690, USA; Horticultural Sciences Department, Plant Molecular and Cellular Biology Program, University of Florida, Gainesville, FL 32611-0690, USA; Horticultural Sciences Department, Plant Molecular and Cellular Biology Program, University of Florida, Gainesville, FL 32611-0690, USA

## Abstract

Functional and architectural diversification of transcription factor families has played a central role in the independent evolution of complex development in plants and animals. Here, we investigate the role of architectural constraints on evolution of B3 DNA binding domains that regulate plant embryogenesis. B3 domains of ABI3, FUS3, LEC2 and VAL1 proteins recognize the same *cis*-element. Complex architectures of ABI3 and VAL1 integrate cis-element recognition with other signals, whereas LEC2 and FUS3 have reduced architectures conducive to roles as pioneer activators. In yeast and plant *in vivo* assays, B3 domain functions correlate with architectural complexity of the parent transcription factor rather than phylogenetic relatedness. In a complex architecture, attenuated ABI3-B3 and VAL1-B3 activities enable integration of cis-element recognition with hormone signaling, whereas hyper-active LEC2-B3 and FUS3-B3 over-ride hormonal control. Three clade-specific amino acid substitutions (β4-triad) implicated in interactions with the DNA backbone account for divergence of LEC2-B3 and ABI3-B3. We find a striking correlation between differences in *in vitro* DNA binding affinity and *in vivo* activities of B3 domains in plants and yeast. Our results highlight the role of DNA backbone interactions that preserve DNA sequence specificity in adaptation of B3 domains to functional constraints associated with domain architecture.

## INTRODUCTION

In plant and animal lineages that have independently evolved embryos (1), key gene regulatory networks that underlie complex development have evolved through functional diversification within families of related transcription factors (2). Functional diversification is facilitated by the typically modular domain architectures of transcriptional regulators. In plants, transcription factors that form a network controlling embryo formation and subsequent transition to the vegetative development share a conserved B3 DNA binding domain (3).

In the B3 network of Arabidopsis, the AFL (ABI3, FUS3 and LEC2) B3 domain proteins primarily function as activators that promote embryogenesis (3), whereas VAL1-type B3 proteins repress the AFL network prior to germination of the seed enabling a transition to vegetative development (4,5). While the B3 domains of AFL activators (3) and VAL repressors bind specifically to the same Sph/RY cis-element (6–12), their distinct roles in the network are differentiated in part at the level of protein domain architecture. ABI3 and VAL1 have complex, multiple domain architectures that physically integrate B3 recognition of the Sph/RY cis-element with hormonal signals (3,13,14) and chromatin marks (3,6,7,11,15,16), respectively, whereas LEC2 and FUS3 have architectures of reduced complexity conducive to their roles as pioneer activators (9,17–23). Orthologs of VP1/ABI3 and VAL1 type proteins occur in all sequenced land plant genomes (18,19), whereas FUS3 and LEC2 genes are restricted to seed plants and rosid clade of angiosperms, respectively (19). This phylogenetic relationship is consistent with FUS3 and LEC2 proteins having evolved via truncation of VP1/ABI3 type ancestors (18,19). While the DNA sequence specificities of B3 domains in VAL1 and AFL proteins are conserved, the contributions of intrinsic differences in B3 domain properties to functional diversification in the B3 transcription factor network have not been systematically addressed.

The AFL transcription factors function at different times in embryogenesis. LEC2 initiates activation of the B3 network during early embryogenesis several days prior to a peak in FUS3 expression (17–23). During late embryogenesis, ABI3 in turn couples the network to abscisic acid (ABA) signaling to promote embryo maturation (13,14,24,25). Protein-protein interactions mediated by the N-terminal COAR domain of VP1/ABI3 orthologs physically couple recognition of the Sph/RY cis-element by the B3 domain to activities of b-ZIP transcription factors that mediate ABA signaling (13,14,26). To dissect COAR- and B3-domain-dependent functions of VP1/ABI3, we developed a transgenic Arabidopsis assay based on maize VP1 (27). In Arabidopsis, a ubiquitously expressed *VP1* transgene (*Pro35S:VP1*) (i) complements the green-seed and desiccation-intolerant seed phenotypes of the *abi3–6* null mutant, and (ii) confers ABA-dependent, ectopic induction of the normally seed specific *Cruciferin-C* (*CRC*) gene in vegetative tissues. *CRC* is a well characterized direct target of ABI3 containing multiple Sph/RY motifs in its promoter (24). Crucially, ABA-dependent induction of *CRC* in vegetative tissues requires both COAR and B3 domains of VP1, whereas the COAR domain alone is sufficient for rescue of *abi3–6* seed maturation phenotypes (28,29).

Here we show that intrinsic differences in activities of ABI3-B3, VAL1-B3, FUS3-B3 and LEC2-B3 domains, measured both in yeast and in a transgenic plant assay that captures integration with abscisic acid (ABA) signaling in a complex architecture setting, reflect a pattern of B3 domain adaptation to complex and reduced protein architectures. In the complex VP1 architecture, phylogenetically distant VAL1-B3 and ABI3-B3 are functionally equivalent in supporting ABA-dependent activation of *CRC* in transgenic plants, whereas in the same context phylogenetically less distant LEC2-B3 and FUS3-B3 are hyper-active causing ABA-independent induction of *CRC*. We identify a clade-specific triad of amino acid substitutions (β4-triad) implicated in electrostatic interactions with the DNA backbone as the structural basis for functional divergence of closely related LEC2-B3 and ABI3-B3 domains both *in vivo* and *in vitro*. We find a striking linear relationship between differences in DNA binding free energy and *in vivo* activities of B3 domains in plant and yeast systems. We propose that selection for attenuated DNA binding affinity is essential for integration of domain functions in transcription factors with complex architectures. LEC2-B3 and FUS3-B3 independently acquired high-DNA binding affinities that facilitate autonomous recognition of the Sph/RY cis-element. In this way, B3 domain adaptation to architectural constraints is enabled by structural variation in interactions with the DNA backbone that alter DNA binding affinity while preserving base-specificity.

## MATERIALS AND METHODS

### Structural analysis and modeling

Structural models of the ABI3 B3 domain bound with DNA were made by MODELLER v9.20 (30) using crystal structures of VAL1 (PDB ID: 6J9A, 6FAS) and FUS3 B3 (PDB ID: 6J9B) complexed to Sph/RY DNA as templates. Fifty replicate structural models were constructed for each template. The best model was selected based on the objective function score (30). Structure analyses and molecular graphics were done in UCSF Chimera (31) (https://www.cgl.ucsf.edu/chimera).

### Phylogenetic analysis

Phylogenetic analyses were performed using online tools hosted at NGPhylogeny (https://ngphylogeny.fr/). Multiple protein sequence alignments of B3 domains were made using MAFFT (32) and maximum-likelihood phylogenetic trees were generated using PHYML (33). Bootstrap values were calculated using BOOSTER (34).

### Yeast one hybrid (Y1H) assays

To produce the WT AFL and VAL Y1H effector plasmids, the B3 domains of ABI3, FUS3, LEC2, VAL1, VAL2 and VAL3 were amplified by PCR (PrimeSTAR TM HS DNA Polymerase, TaKaRa) using the plasmids carrying the full-length cDNA of each gene as template. The corresponding PCR products were cloned into the pGADT7 vector (Clontech, CA) between the ClaI and SacI sites, which generated N terminal GAL4 activation domain (GAL4 AD) fusion for each Y1H effector. pGADT7 has the *LEU2* selection marker in yeast.

The B3 domain mutant effector plasmids were produced by using AFL WT B3 domain effector plasmids as templates and then introducing point mutations at the 3 targeted residues (residues 64, 66 and 69), respectively. All site-directed mutagenesis reactions were performed by following the instructions in the QuikChange Lightning Multi Site-Directed Mutagenesis Kit (Agilent Technologies, Stratagene, CA, USA). Primers used for PCR and site-directed mutagenesis reactions are indicated in Supplementary Table S1. The Y1H reporter plasmid pHISi-1-Sph2 was constructed by inserting a 40 bp fragment that contains Sph dimer (named as Sph2) into the pHISi-1 vector (with *HIS3* selection marker): 5′-GATCATGCATGGACGACACGGATCATGCATGGACGACACG-3′ (Sph underlined). The pHISi-1 vector was provided by the Matchmaker one-hybrid kit (Clontech, CA, USA). The 40 bp promoter sequence originated from the maize *C1* gene promoter (35). All the constructs were verified by sequencing. To generate the reporter strain Sph2-YM4271, Y1H reporter plasmids pHISi-1-Sph2 were linearized by XhoI and stably integrated into the non-functional *HIS3* locus of Yeast YM4271 by homologous recombination following the manufacturer's instructions (Clontech, CA). To control basal expression of *HIS3* reporter gene, Sph2-YM4271 cells were grown on synthetic dropout (SD)-His media supplemented with the inhibitor 3 mM 3-amino-1,2,4-triazole (3-AT; Sigma). All Y1H effector plasmids were separately transformed into the Sph2-YM4271 reporter strain and plated on SD/-Leu plates. The plates were incubated at 30°C for 4 days to screen the positive transformations.

To test the interaction of effectors with the Sph2 reporter, transformed yeast reporter cells were cultured overnight in SD-Leu liquid medium. Cells were then pelleted, washed, and resuspended in water. OD_600_ was measured using a microplate reader Epoch (Biotek). Then the concentration of each transformed yeast cell culture was normalized to OD_600_ = 0.04. Ten-fold serial dilutions of each normalized culture were prepared. A 3 μl sample from each culture in the dilution series was spotted on SD/-Leu, SD/-Leu/-His, and SD/-Leu/-His plus 3mM 3-AT plates, respectively, and incubated at 30°C for 4 days.

For yeast growth curves, cell cultures that normalized to a uniform OD_600_ (OD_600_ = 0.04) in 1.5 ml of SD/-Leu/-His liquid medium (plus 3 mM 3-AT) were incubated in a 12-well microplate (Thermo Scientific) at 30°C for 96 h. The OD_600_ was measured in a microplate reader Epoch (Biotek) at 12 h intervals. Three replicates of each treatment were used in these experiments.

### Construction of VP1::B3 chimeral transgenic constructs

The 35S-VP1 construct was created previously in our lab (27). The full length *VP1* cDNA that includes XbaI and XhoI sites at the 5′- and 3′-ends, respectively, was amplified using the VP1-XbaI/VP1-XhoI primer pair and 35S-VP1 plasmid as a template. The resulting PCR product was sub-cloned into pCR4-TOPO vector (Invitrogen) to create VP1-TOPO. AFL B3 and VAL1 chimeral transgene (*Pro35S:VP1::ABI3-B3*, *Pro35S:VP1::FUS3-B3*, *Pro35S:VP1::LEC2-B3* and *Pro35S:VP1::VAL1-B3*) constructs were produced by the following several steps: (i) EcoRV and SacI restriction sites that flanked the B3 domain were introduced into the VP1-TOPO plasmid by site-directed mutagenesis (Agilent Technologies, CA, USA); (ii) the plasmids created in step 1 and the respective PCR amplified AFL and VAL1 B3 domain sequences that carrying EcoRV and SacI at both ends were digested with EcoRV and SacI. The restriction fragments containing the VP1-TOPO plasmid backbone and the AFL and VAL1 B3 domain sequence were purified and ligated together; (iii) the restriction sites at the swap borders were then restored to the original sequence by site-directed mutagenesis and (iv) AFL B3 and VAL1 chimeral transgenes (*VP1::ABI3-B3*, *VP1::FUS3-B3*, *VP1::LEC2-B3* and *VP1::VAL1-B3*) in TOPO plasmid were digested with XbaI/XhoI and ligated into the XbaI/SalI sites of the transformation vector pCAMBIA1300, which placed the 35S promoter upstream of the transgene and attached a GFP tag to C-terminus of the hybrid protein (Figure 3A). The mutant chimeral transgenes were created by site-directed mutagenesis using corresponding templates in the TOPO plasmids and ligated to pCAMBIA1300. The Primers used for PCR and site-directed mutagenesis reactions are indicated in Supplementary Table S2. All the constructs were verified by sequencing.

### Generation of transgenic plants

The wild type and mutant chimeral transgenic constructs were used to transform to agrobacterium strain GV3101, and plated on YEP media (10 g yeast extract, 10 g Bacto peptone and 5 g NaCl per liter, pH 7.0) containing 50 mg/l kanamycin (Kan) and 25 mg/l gentamicin sulfate. Agrobacterium-mediated transformation of *abi3–6* mutant Arabidopsis plants was performed by using the floral-dip method (36). T1 seeds were harvested about 3–4 weeks after transformation. T1 seeds were sterilized and stratified at 4°C in dark for 3 days. To screen for hygromycin resistance, stratified T1 seeds were plated on MS media containing 1X Murashige and Skoog salt, 0.05% MES, 1% sucrose sterilized by filtration and 0.15% of phytagel (Sigma) supplemented with 25 mg/l hygromycin and 200 mg/l carbenicillin (to suppress the Agrobacterium). Plated seeds were grown at 23°C for 1 week under continuous light. Plants that exhibited rapid relative growth rates were selected as candidate transgenic seedlings and grown on the MS media containing 25 mg/l hygromycin for an additional week. The surviving hygromycin resistant T1 seedlings that had good root formation were transferred to soil using plant growth conditions described previously (5). T2 seeds of independent lines that segregated approximately 3:1 brown: green colored seeds were screened again for hygromycin resistance. The homozygous transgenic seeds were screened in the T3 generation by scoring seed color (all brown) as well as 100% hygromycin resistance on selection medium.

### Gene expression analysis

Detached leaf tissue of 14-day-old T1 transgenic seedlings were incubated 24 h on MS and MS plus 5 μM ABA, respectively. *Col-0* WT and *abi3–6* seedlings were used as controls in this assay. Total RNA was extracted from the leaf samples, or whole T1 seedlings in cases of abnormal morphology, using the plant miniRNA kit (Zymo research). RNA concentration was quantified by Nanovue Plus (GE Healthcare). RQ1 RNase-Free DNase (Promega) was used to remove genomic DNA in total RNA of each sample. Quantitative Real-Time (qRT-PCR) assay was carried out with a Power SYBR Green RNA-to-Ct 1-Step Kit (Applied Biosystems) on a StepOnePlus system (Applied Biosystems). An absolute quantification method was used to analyze gene expression. Standard curves were constructed using plasmids containing the target gene sequences. TUB2 (AT5G62690) was used as an endogenous control in the RT-PCR. Transgene expression was quantified using primers that targeted the NOS terminator region common to all transgenes. The primers used for RT-PCR and quantitative PCR are listed in Supplementary Table S2.

### *In vitro* DNA binding assays

The AFL and VAL1 B3 domains were sub-cloned by using *VP1::B3* chimeral transgenic constructs (Figure 3A and Supplementary Figure S10) as templates. A common pairs of primers B3-BamHI/B3-EcoRI (Supplementary Table S3) were used to incorporate BamHI and EcoRI sites for cloning into pGEX-2T (Phamacia Inc., Uppsala, Sweden). Constructs were verified by sequencing. All GST-B3 fusion proteins were expressed in *Escherichia coli BL21(DE3)* cells and were purified by gluthathione-sepharose affinity chromatography according to the manufacturer's recommendations (GE Healthcare). The proteins were eluted in elution buffer (20 mM reduced glutathione, 100 mM Tris–HCl, pH 8.0). The purified GST fusion proteins were visualized by sodium dodecyl sulphate-polyacrylamide gel electrophoresis (SDS-PAGE). Protein concentrations were measured via bradford assay (37). Sph2 probe is 5′ biotin labeled (synthesized by IDT) and is shown in Supplementary Table S3.

B3-DNA binding was measured in a label-free in vitro kinetics assay at pH 7.0 using Octet Qke system (Pall ForteBio) (38). The 1× Kinetics Buffer (Pall ForteBio) was used as running buffer. The experimental steps are: after a 5 min initial baseline step, 0.05 μM biotinylated Sph2-probe was loaded onto streptavidin biosensors for 5 min until reach saturation. Probes were then quenched by 25 μg/ml biocytin for 2 min. After a 2 min baseline step, each probe was then exposed to B3 protein at increasing concentrations for 15 min at association step, followed by 30 min dissociation step. Changes in the number of molecules bound to the biosensor causes a shift in the interference pattern that is measured in real time (Pall ForteBio application note 14). Measurements were taken from one experimental replicate.

### Analysis of biolayer interferometer DNA binding data

In biolayer interferometry DNA binding experiments performed on the Octet Qke instrument, dissociation time-course data for B3 domains included distinct fast and slow dissociating components. To resolve the fast- and slow-dissociating fractions of total binding, biolayer interferometry time course data for dissociation of B3-DNA complexes were fit to a two-component exponential model using the non-linear-least-square (nls) function in R (R-project.org). In the model equation, *v* = b* *v*_0_* exp(–*t* * *k*_fast_) + (1 – *b*) * *v*_0_ * exp(–*t* * *k*_slow_), *v* is the concentration of protein–DNA complex at time *t* (s) relative to the initial concentration of bound complex (*v*_0_), *b* is the fraction of *v*_0_ having a fast dissociation rate (off-rate constant *k*_fast_) while the remaining tightly bound fraction dissociates with off-rate *k*_slow_ (Supplementary Table S6). The off-rate constant estimates were then used to constrain modeling of the full reaction time-courses in COPASI (39) using a model that included independent low- and high-affinity binding sites. This model was selected for comparison of B3 domain DNA binding properties based on (i) acceptable goodness of fit based on the values of the COPASI parameter estimation objective function and visual assessment across the five B3 domains analyzed (compare two-site and one-site fits in Supplementary Figures S13A, B) and (ii) on the strong correlation of *K*_d1_ estimates for the lower-affinity site with *in vivo* activity (Figure 6). Values for the respective on-rate constants (*k*_on1_ and *k*_on2_) for low- and high-affinity sites (Supplementary Table S7) were estimated using the parameter estimation tool (evolutionary programing method) of COPASI simultaneously fitting full reaction time courses at three protein concentrations. LEC2-B3, ABI3-B3 and LEC2-B3 RRP and ABI3-B3 KKS substitution mutants were analyzed at 1E−07 M, 2E−07 M and 4E−07 M B3 protein. FUS3-B3 parameters were fit using 0.2E−07 M, 0.4E−07 M and 1.0E−07 M protein reactions. *K*_d1_ and *K*_d2_ dissociation constants in Table 1 were calculated from ratios of the respective off and on rate constants (*K*_d1_ = *k*_fast_/*k*_on1_; *K*_d2_ = *k*_slow_/*k*_on2_).

**Table 1.** Properties^a^ of low- and high-affinity *in vitro* DNA binding sites

	*K*_d1_	*K*_d2_	Δ*G*°_1_
	(M)	(M)	(kJ mol^−1^)
**ABI3-B3**	2.00E−06 (2.95E−07)^b^	2.62E−08 (5.13E−10)	−32.51 (0.34)
**LEC2-B3**	3.66E−07 (5.11E−08)	2.23E−08 (4.74E−09)	−36.72 (0.32)
**ABI3-B3 KKS**	4.99E−07 (7.35E−08)	3.48E−08 (3.34E−09)	−35.95 (0.34)
**LEC2-B3 RRP**	9.00E−07 (2.05E−07)	1.86E−08 (6.12E−09)	−34.49 (0.51)
**FUS3-B3**	2.63E−07 (4.94E−09)	4.16E−09 (4.77E−10)	−37.54 (0.05)

^a^ *K*_d1_ = *k*_fast_/*k*_on1_; *K*_d2_ = *k*_slow_/*k*_on2_ (see Supplementary Table S3 and S4). Δ*G*°_1_ = −*RT* ln(1/*K*_d1_).

^b^Values in parentheses are standard errors calculated by propagation of errors for on- and off-rate constants (Supplementary Table S6 and S7).

## RESULTS

### Architectural diversification of B3 domain transcription factors

The VAL1 and VP1/ABI3 type B3-domain transcription factors with characteristic domain compositions occur in diverse vascular plant genomes indicating that these architectures arose in concert with evolution of land plants (embryophytes) consistent with their fundamental roles in plant embryo development. By contrast, FUS3- and LEC2-type transcription factors, which have similar architectures of reduced complexity, are restricted to seed plants and to the rosid clade of the angiosperms (18,19), respectively, suggesting that they evolved via truncation of VP1/ABI3-like ancestors.

#### Evidence for architectural constraints on B3 domain evolution

**Figure 1.**
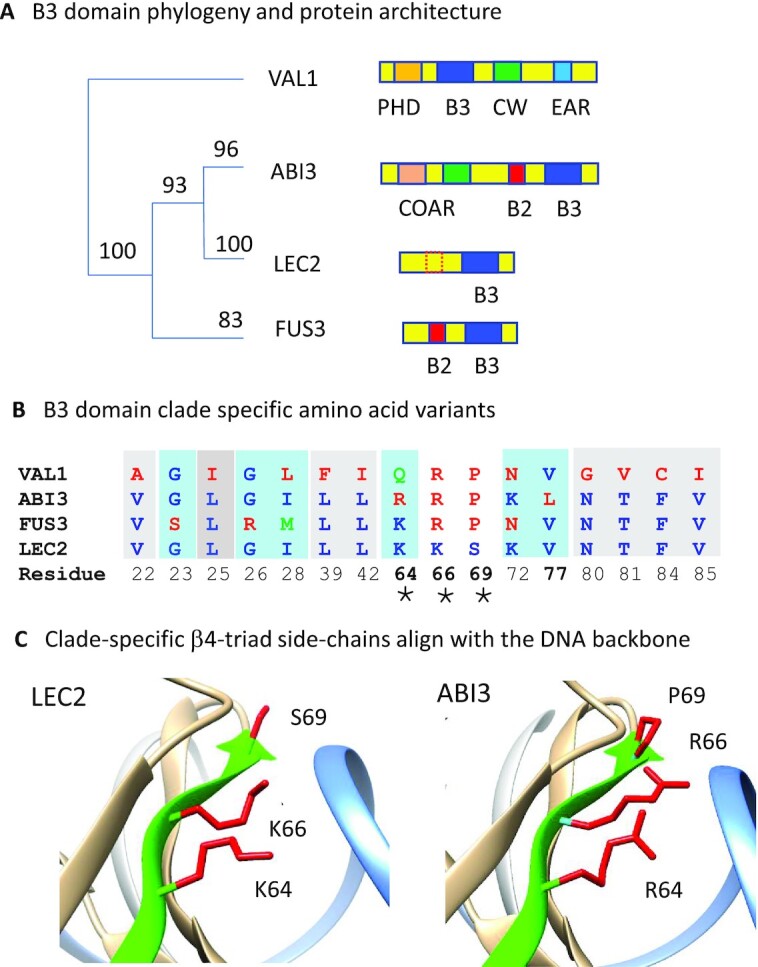
A structural basis for functional differentiation of LEC2 and ABI3 B3 domains. (**A**) B3 domain phylogeny and protein domain architectures of ABI3, FUS3, LEC2 and VAL1 transcription factors. The tree is adapted from a phylogenetic analysis of B3 domain sequences from diverse orthologs of ABI3, FUS3, LEC2 and VAL1 (Supplementary Figure S1). Multiple protein sequence alignments and maximum-likelihood tree were constructed using MAFFT (32) and PhyML (33) with bootstrap support based on BOOSTER (34). (**B**) B3 domains are distinguished by clade-specific structural variants at 16 amino acid positions. Clade-specific variant positions meet two criteria: (i) positions include an amino acid substitution in at least one B3 clade, and (ii) all substitutions observed at that position are strictly conserved within each of the four clades. Here and elsewhere, amino acid positions are numbered uniformly according to the sequence alignment shown in Supplementary Figure S2. Eight clade-specific variants that distinguish VAL1 from the three AFL B3 clades are shaded in grey background. Six clade-specific variant positions that distinguish FUS3 B3 from ABI3 B3 are shaded light blue. Four clade-specific positions that distinguish ABI3 and LEC2 B3 sequences are highlighted with bold numbers. The latter include the β4-triad (marked by *), comprised of variants at positions 64, 66 and 69 located in beta sheet strand 4 (Supplementary Figure S2). (**C**) Locations of β4-triad amino acids in the B3 domain align with the DNA backbone. Images showing β4-triad amino acid side-chains (colored red) in LEC2 B3 (6J9C.pdb) (9) and a structural model of ABI3 B3 based on FUS3 (6J9B.pdb) (9) were generated with Chimera (31). A ribbon depicting the β4 strand is colored green, and proximal DNA backbone strand is colored light blue. The ABI3 B3 model was constructed by MODELLER (30).

A phylogenetic analysis of B3 domain sequences was undertaken to further resolve relationships among the B3 domains of VAL1 and the three AFL proteins (Figure 1A and Supplementary Figure S1). A salient feature of the B3 domain tree is that the VP1/ABI3 clade, which spans ∼400 MY of land plant evolution, is clearly separated from the putatively derived FUS3-B3 and LEC2-B3 clades. This separation is noteworthy because if (i) FUS3 and LEC2 proteins were indeed derived from ancestors in the VP1/ABI3 clade, and (ii) conservation of DNA binding sequence specificity were the sole functional constraint on B3 domain evolution, then we would instead expect FUS3-B3 and LEC2-B3 clades to be nested within the VP1/ABI3 clade consistent with their later origins within the seed plants. To account for the observed separation of VP1/ABI3-B3 from FUS3-B3 and LEC2-B3 clades, we hypothesized that evolution of B3 domains in VP1/ABI3 type proteins is subject to additional functional constraints imposed by a complex architecture. A key prediction of that hypothesis is that B3 domains derived from proteins with simple and complex architectures, respectively, are not functionally equivalent.

#### Evidence for independent origins of LEC2 and FUS3

The B3 domain phylogeny further indicated that LEC2-B3 is more closely related to VP1/ABI3-B3 clade than to FUS3-B3. A parsimonious interpretation is that LEC2 and FUS3 diverged independently from VP1/ABI3-type progenitors through loss of the N-terminal COAR domain (Figure 1A and Supplementary Figure S1). The FUS3-B3 clade evidently originated early in the seed plant lineage, whereas LEC2-B3 arose more recently in the rosid clade of angiosperms (flowering plants). Hence, the comparatively recent separation of LEC2-B3 and ABI3-B3 domains afforded an opportunity to identify intrinsic differences in B3 domain structure associated with functional diversification of LEC2 and ABI3 transcription factors.

### A structural basis for functional differentiation of LEC2 and ABI3 B3 domains

#### Identification of clade-specific structural variants

To identify conserved structural features that distinguish of LEC2-B3 and ABI3-B3 domains, variable positions in B3 multiple protein sequence alignments were classified as clade-specific if all amino acid substitutions observed at that position were strictly conserved within each of the four B3 clades (Figure 1B and Supplementary Figure S2). Strikingly, three of four clade-specific amino acid variants that distinguish ABI3-B3 and LEC2-B3 domains are clustered in beta-strand β4 of the protein structure (Figure 1B, C). In B3-DNA complexes (8,9), the C-terminal end of β4 approaches the DNA backbone at an oblique angle (Figure 1C, green ribbon) aligning side chains of the β4-triad amino acids with the phosphate backbone of DNA.

### Analysis of *in vivo* B3 domain functions in yeast

**Figure 2.**
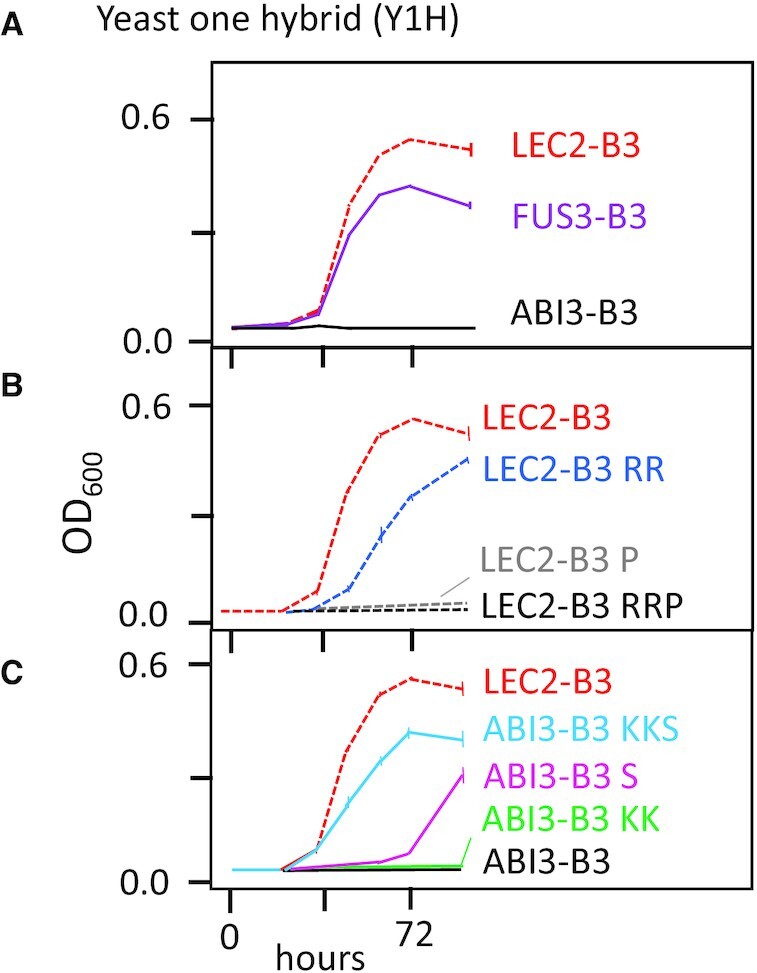
Functional analysis of B3 domains in yeast. A yeast-one-hybrid (Y1H) assay for functional analysis of B3 domains is based on activation of a HIS selectable marker controlled by a minimal promoter containing a dimer of the Sph/RY cis-element recognized by AFL and VAL1-B3 domains (3,6–11) (Supplementary Figure S3). Activities of B3 effector proteins were quantified by measuring HIS dependent growth of yeast cultures (OD_600_, optical density at 600 nm) over a 96-hour time course. (**A**) Effector proteins containing LEC2-B3 (dashed red line) and FUS3-B3 (solid violet line) domains supported growth on his selective media (Supplementary Figure S3), whereas ABI3-B3 (solid black) and VAL1-B3 (Supplementary Figure S3) were in-active in yeast. (**B**) Substitutions of LEC2-B3 β4-triad residues with ABI3-B3 variants partially (LEC2-B3 RR, dashed blue) or fully (LEC2-B3 P, dashed grey, and LEC2-B3 RRP, dashed black) abolished activity in yeast. (**C**) Reciprocal substitutions of β4-triad residues in ABI3-B3 with the LEC2-B3 variants (ABI3-B3 S, solid magenta, and ABI3-B3 KKS, solid light blue) conferred partial activity in yeast, whereas the ABI3-KK double substitution (solid green) was not active. Error bars indicate standard error of the mean for three replicates.

To quantify *in vivo* activities of B3 domains in isolation, we adapted a yeast one hybrid (Y1H) assay (21) based on a minimal *HIS3* promoter containing tandem copies of the Sph/RY *cis*-element (Figure 2A and Supplementary Figure S3). In this assay, GAL4AD::B3 effector proteins containing LEC2-B3 and FUS3-B3 domains supported growth of transformed yeast cells on selective agar media (Supplementary Figure S3) as well as in liquid culture (Figure 2A). On both media, GAL4AD::LEC2-B3 was more effective in promoting growth than GAL4AD::FUS3-B3. By contrast, GAL4AD::ABI3-B3 (Figure 2A) and GAL4AD::VAL1-B3 effectors (Supplementary Figure S3) did not support yeast growth in the one-hybrid assay.

#### Functional analysis of β4-triad structural variants in yeast

To determine whether β4-triad residues account for the qualitative difference *in vivo* activities of LEC2-B3 and ABI3-B3 domains measured in yeast, we analyzed a series of reciprocal substitutions that interconvert β4-triad amino acids in the ABI3-B3 and LEC2-B3 domains (Figure 2B, C). In LEC2-B3 RR, the combined K64R and K66R substitutions reduced yeast growth rate compared to wild-type LEC2-B3, whereas the S69P single mutant (LEC2-B3 P) and LEC2-B3 RRP triple mutant completely abolished HIS3 dependent growth (Figure 2B). Conversely, as shown in Figure 2C, introduction of the LEC2 β4-triad variants in ABI3-B3 KKS resulted in a qualitative gain of effector activity (about 60% of LEC2-B3 activity) compared to the inactive wild type ABI3-B3. The P69S substitution (ABI3-B3 S) alone conferred weak activity, whereas combined R64K and R66K substitutions (ABI3-B3 KK) had no effect.

### Analysis of *in vivo* B3 domain functions in Arabidopsis

#### Functional analysis of VAL1-B3 and AFL-B3 domains in a complex architecture setting

**Figure 3.**
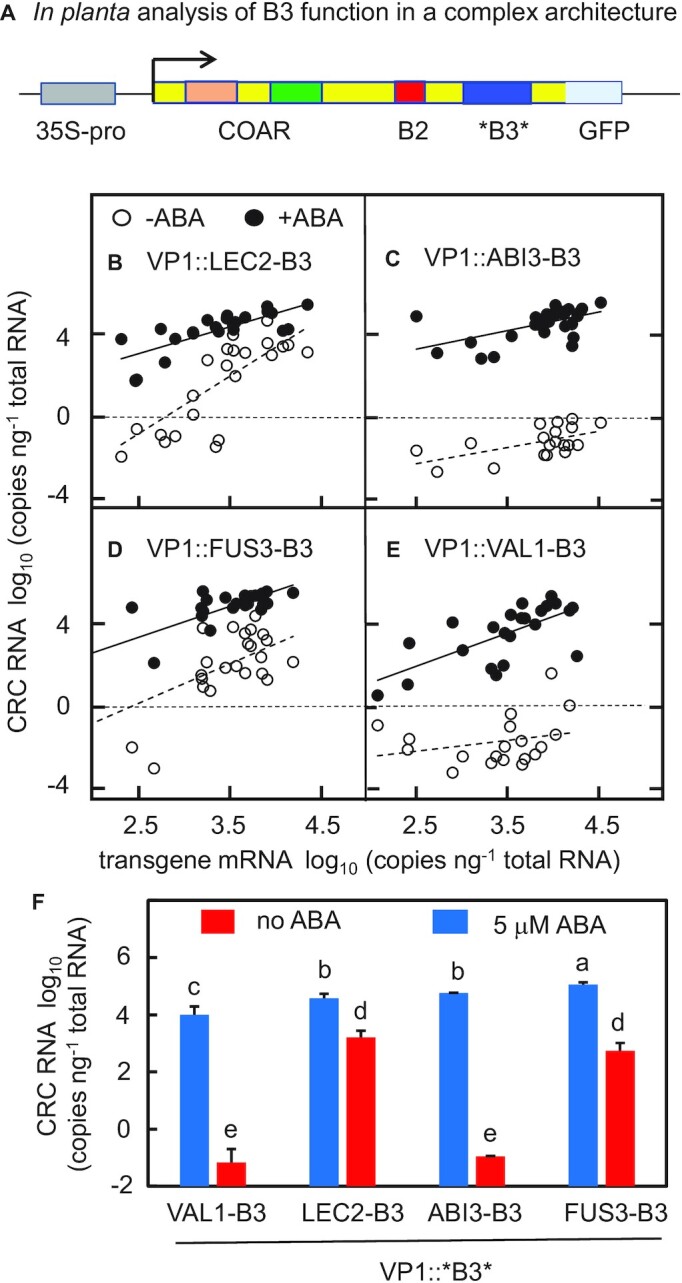
*In vivo* analysis of B3 domain functions in a complex architecture setting. (**A**) To quantify B3 capacities to interoperate with ABA signaling mediated by the COAR domain in Arabidopsis, ABI3-B3, LEC2-B3, FUS3-B3 and VAL1-B3 domain sequences were swapped into a heterologous *35S-promoter-VP1* transgene (junctions indicated by *). This transgenic assay takes advantage of the observation that constitutive expression of maize *VP1* complements the green embryo and desiccation intolerant seed phenotypes of the Arabidopsis *abi3–6* mutant and conditions ectopic, ABA induction of the endogenous *CRC* gene in leaves (27). ABA induction of *CRC* requires both COAR and B3 functions (28,29). (B−E) Activation of *CRC* by VP1::LEC2-B3, VP1::ABI3-B3, VP1::FUS3-B3 and VP1::VAL1-B3, respectively, in transgenic seedlings incubated on media containing 5 μM ABA (solid circles, solid regression line) and no ABA (open circles, dashed regression line). RNA levels of the indicated *VP1::B3* transgene and *CRC* were quantified in individual transformed T1 seedlings by qPCR and expressed as log10 (RNA copies ng^−1^ total RNA). Horizontal dashed lines at log_10_ = 0 are included for reference. All four B3 domains supported ABA-dependent induction of *CRC* in leaves (solid circles). *VP1::LEC2-B3* (**B**) and *VP1::FUS3-B3* (**D**) caused strong activation of *CRC* in absence of ABA, whereas activation of *CRC* by *VP1::ABI3-B3* (**C**) and *VP1::VAL1-B3* (**E**) showed strict dependence on ABA signaling (open circles). (**F**) Comparisons of ABA-dependent (blue) and ABA-independent (red) *CRC* activation in seedlings with high-transgene expression (>3.5 log_10_ copies of transgene RNA ng^−1^ total RNA). *CRC* activation is expressed as log10 CRC RNA copies ng^−1^ total RNA relative to a baseline of 0.01 CRC RNA copies per ng total RNA. Differences between values assigned non-identical lowercase letters (a, b, c, d, e) were statistically significant based on pairwise *t*-tests (*P* < 0.05). Error bars indicate standard error of the mean.

In developing embryos of plants, protein-protein interactions mediated by the N-terminal COAR domain of VP1/ABI3 proteins physically couple recognition of the Sph/RY cis-element by the C-terminal B3 domain to activities of b-ZIP transcription factors that mediate abscisic acid (ABA) signaling (13,14,26). To test interoperability of different B3 domains with the COAR domain we used VP1, the maize ortholog of ABI3, as a heterologous domain-swap host for testing and comparing B3 domain function in transgenic Arabidopsis (Figure 3A and Supplementary Figure S4 and S5). As shown previously (27) a ubiquitously expressed *VP1* transgene (*Pro35S:VP1*) (i) complements the green-seed and desiccation-intolerant seed phenotypes of the *abi3–6* null mutant (Supplementary Figure S4), and (ii) confers ABA-dependent, ectopic induction of the normally seed-specific ABI3 target *CRC* (27,28) in vegetative tissues (Supplementary Figure S5). Crucially, ABA-dependent induction of *CRC* in vegetative tissues requires both COAR and B3 domains of VP1, whereas COAR function alone is sufficient for rescue of the desiccation intolerant seed phenotype of *abi3* (28,29). As a measure of B3 activity, we quantified expression of both the *VP1::B3* transgene and endogenous *CRC* gene in detached leaves of individual 14-day-old T1 transgenic seedlings using qPCR following 24h incubation on MS media with or without a 5 μM ABA treatment (Figure 3B−E and Supplementary Figure S5). Due to position effect variation among individual transformants, the analysis spanned a >300-fold dynamic range of transgene expression (∼2.5 log units, Figure 3B−E, x-axes). To confirm that ectopic induction of *CRC* in the transgenic assay required B3 domain activity (27), K15R loss-of-function mutations were tested for each B3 domain as negative controls (Supplementary Figure S6).

#### ABA-dependent and ABA-independent activities of B3 domains

As shown in Figure 3B−E (solid circles), in the VP1 context each of the four B3 domains supported strong ABA-dependent induction of *CRC* (>4.0E+04 mRNA copies per ng RNA) though quantitative differences were evident. As shown in Figure 3F (blue bars), at high transgene dosage levels VP1::FUS3-B3 exhibited the greatest capacity for ABA-dependent activation of *CRC*, whereas activities of VP1::ABI3-B3 and VP1::LEC2-B3 were intermediate and higher than VP1::VAL1-B3.

By contrast, B3 domains exhibited striking qualitative differences in their capacities for activation *CRC* in absence of ABA (Figure 3B-E, open circles, Figure 3F, red bars). VP1::LEC2-B3 and VP1::FUS3-B3 caused strong activation of *CRC* in the absence ABA signaling (Figure 3F, red bars), whereas *CRC* activation by VP1::ABI3-B3 and VP1::VAL1-B3 transgenes showed strict ABA dependence (<0.1 *CRC* RNA copy per ng total RNA, except in 2 seedlings with very high VP1::VAL1-B3 expression). ABA-independent activation of *CRC* by VP1::LEC2-B3 and VP1::FUS3-B3 increased with transgene expression (Figure 3B, D) reaching maximum levels of *CRC* expression that were one to two log units lower than obtained in ABA treated seedlings (Figure 3F).

#### Ectopic callus phenotypes induced by VP1::LEC2-B3 and VP1::FUS3-B3 transgenes

**Figure 4.**
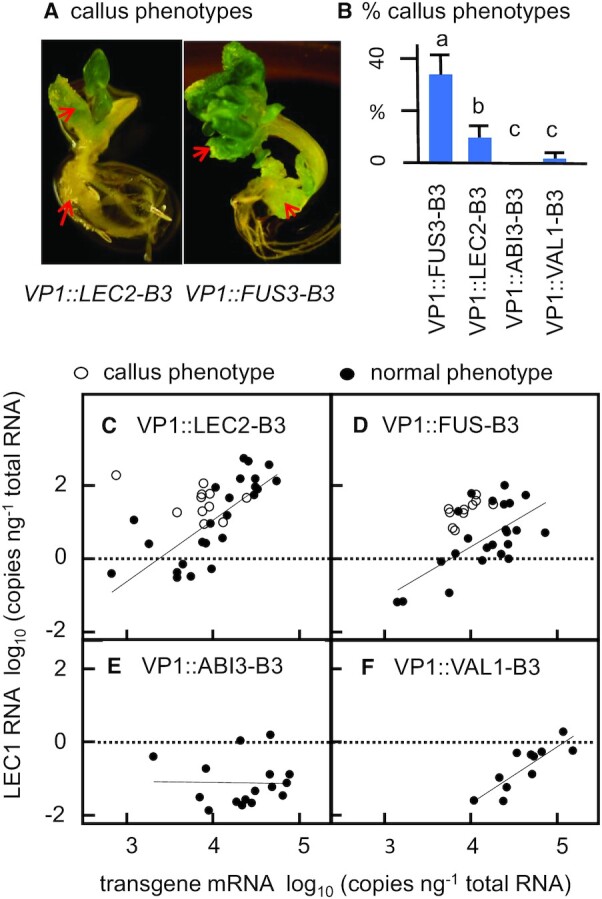
Abnormal seedling phenotypes induced by *VP1::LEC2-B3* and *VP1::FUS3-B3* transgenes. (**A**) Representative images of callus phenotypes observed in a subset of *VP1::LEC2-B3* and *VP1::FUS3-B3* transformants (red arrows, callus). (**B**) Observed frequencies of callus phenotypes in independent transformants of each transgene. Differences between values assigned non-identical lowercase letters were statistically significant based on pairwise t-tests (*P* < 0.05). (**C−F**) Ectopic activation of endogenous *LEC1* in *VP1::LEC2-B3*, *VP1::FUS3-B3*, *VP1::ABI3-B3* and *VP1::VAL1-B3* transformants that had normal (solid circles) and callus phenotypes (open circles). *CRC* expression data from abnormal seedlings were not included in Figure 3.

A subset of *VP1::FUS3-B3* and *VP1::LEC2-B3* transformants produced abnormal seedlings with features resembling embryo callus phenotypes associated with *val* loss of function mutants (4,5) (Figure 4A, Supplementary Figure S7). By contrast, callus induction was rare among *VP1::ABI3-B3* and *VP1::VAL1-B3* transformants (Figure 4B). The frequency of callus phenotypes in *VP1::FUS3-B3* transformants (35%) was 3-fold higher than observed in *VP1::LEC2-B3* seedlings. In *val* loss-of-function mutants, embryonic seedling phenotypes are associated with up-regulation of *LEC1*, a transcription factor implicated in initiation of embryogenesis (4,5). Ectopic activation of *LEC1* was evident in all *VP1::FUS3-B3* and *VP1::LEC2-B3* transformants that exhibited callus phenotypes (Figure 4C, D, open circles) as well as in a majority of transformants with normal seedling phenotypes (Figure 4C, D, solid circles) indicating that induction of *LEC1* was likely necessary but not sufficient for induction of embryonic seedling phenotypes. In line with that conclusion, *LEC1* expression in *VP1::ABI3-B3* and *VP1::VAL1-B3* transformants was consistently low (Figure 4E, F).

### β4-triad differentiation of LEC2-B3 and ABI3-B3 activities in Arabidopsis

**Figure 5.**
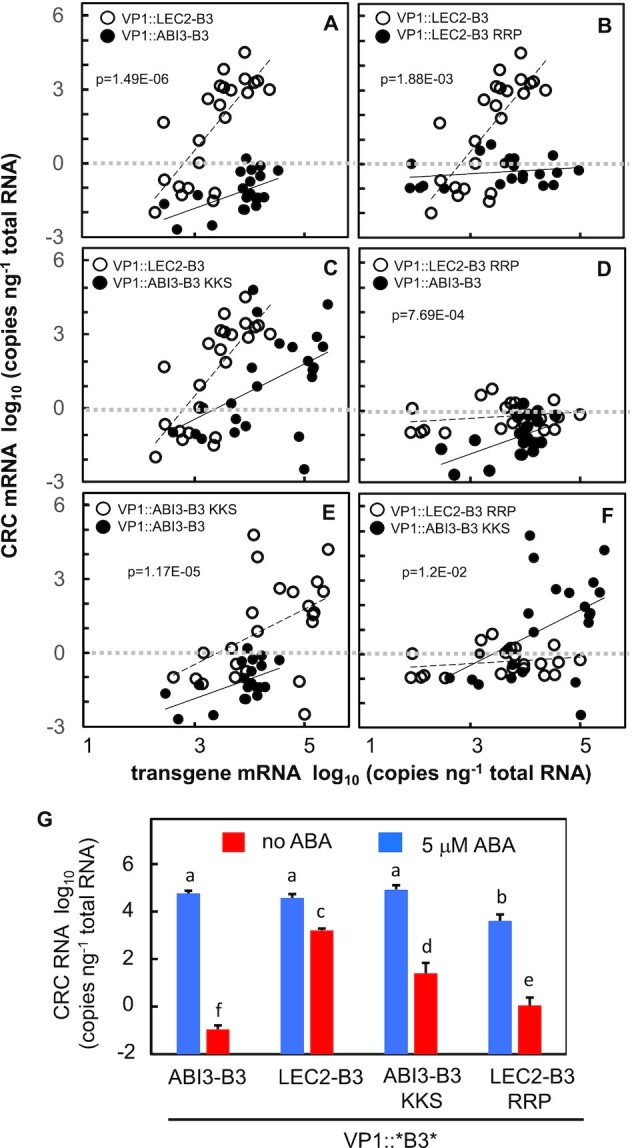
β4-triad effects on *in vivo* activities of ABI3-B3 and LEC2-B3 in plants. (**A**) Comparison of ABA-independent *CRC* activation in seedlings transformed with *VP1::LEC2-B3* (open circles) and *VP1::ABI3-B3* (filled circles). (**B**) Comparison of *VP1::LEC2-B3* (open circles) and *VP1::LEC2-B3 RRP* β4-triad triple substitution (filled circles). (**C**) Comparison of *VP1::LEC2-B3* (open circles) and *VP1::ABI3-B3 KKS* β4-triad triple substitution (filled circles). (**D**) Comparison of VP1::LEC2-B3 RRP β4-triad triple substitution (open circles) and VP1::ABI3-B3 (filled circles). (**E**) Comparison of *VP1::ABI3-B3 KKS* β4-triad triple substitution (open circles) and *VP1::ABI3-B3* (filled circles). (**F**) Comparison of *VP1::LEC2-B3 RRP* (open circles) and *VP1::ABI3-B3 KKS* (filled circles) β4-triad triple substitutions. *P*-values are shown for statistically significant differences between means taken over all levels of transgene expression. Means and pairwise t-test statistics are summarized in Supplementary Table S4 and Supplementary Table S5, respectively. Regression lines for open and filled circles are dashed and solid, respectively. Horizontal dashed lines are drawn at log_10_ = 0 for reference. (**G**) β4-triad effects on ABA-dependent (blue) and ABA-independent (red) activities of *VP1::B3* transgenes *in vivo*. As in Figure 3F, *CRC* activation in seedlings with high transgene expression (>3.5 log_10_ transgene RNA ng^−1^ total RNA) is expressed as log_10_ (CRC RNA copies ng^−1^ total RNA) relative to a baseline of 0.01 CRC RNA copies per ng total RNA. Error bars indicate standard error of the mean.

As noted above, the VP1::LEC2-B3 and VP1::ABI3-B3 had strikingly different capacities for ABA-independent activation of *CRC*, whereas their capacities for ABA-dependent *CRC* induction were similar (Figure 3F and Supplementary Figure S8). To determine whether the qualitative difference in ABA-independent activation of *CRC* was attributable to the clade-specific β4-triad structural variant, we tested effects of reciprocal β4-triad substitutions in VP1::LEC2-B3 and VP1::ABI3-B3 proteins (Figure 5A−F). As shown in Figure 5B and G, the VP1::LEC2-B3 RRP β4-triad substitution had a sharply reduced capacity for ABA-independent activation of *CRC* compared to VP1::LEC2-B3. ABA-dependent *CRC* activation was also impacted though less strongly (Figure 5G, blue bars; Supplementary Figure S8). In these respects, VP1::LEC2-B3 RRP resembled VP1::ABI3-B3 (Figure 5D) though it retained somewhat greater ABA-independent activity (Figure 5D, G). Conversely, the VP1::ABI3-B3 KKS β4-triad substitution exhibited marked higher ABA-independent activation of *CRC* in seedlings in comparison to VP1::ABI3-B3 (Figure 5E) including values at high transgene dosage levels were comparable to the activity of VP1::LEC2-B3 (Figure 5C). By contrast, the ABA-dependent activities of VP1::ABI3-B3 KKS and VP1::ABI3-B3 were similar (Figure 5G and Supplementary Figure S8). Hence, the reciprocal β4-triad substitutions resulted in partial interconversion of LEC2-B3 and ABI3-B3 domain activities in absence of ABA signaling (compare Figure 5A and F). The effects of reciprocal single amino acid substitutions at position 69, ABI3-B3 S and LEC2-B3 P, were intermediate though with increased variation in transgene dosage responses (Supplementary Figure S9; Supplementary Tables S4 and S5).

### Correlation of B3 domain i*n vivo* activity with *in vitro* DNA binding affinity

**Figure 6.**
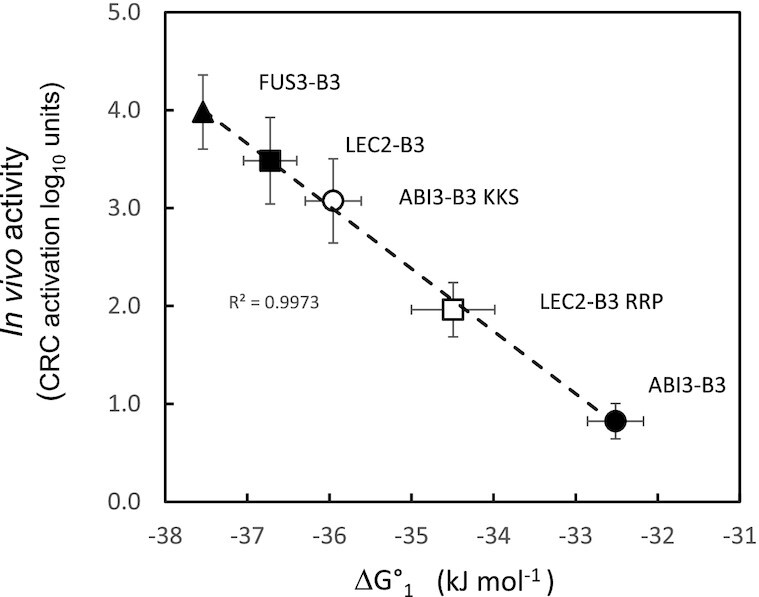
DNA binding free energies of wild type and β4-triad mutant B3 domains correlate with *in vivo* activity. Linear regression of the binding free energy, Δ*G*°_1,_ with ABA-independent activation of *CRC* in transgenic Arabidopsis. *In vivo* activity is expressed as log_10_ units above a baseline of 0.01 copies CRC RNA ng^−1^ total RNA. Means include seedlings with log_10_ (transgene RNA ng^−1^ total RNA) values >2.5 (compare threshold of 3.5 used in Figure 3F and 5G). *R*^2^ was determined by linear regression (dashed lines). ABI3-B3 (solid circle), ABI3-B3 KKS (open circle), LEC2-B3 (solid square), LEC2-B3 RRP (open square) and FUS3-B3 (solid triangle). Horizontal error bars for Δ*G*°_1_ were propagated from standard errors of *K*_d1_ estimates in Table 1, vertical error bars are standard error of the mean for *CRC* expression.

To determine a biochemical basis for the qualitative functional differences of B3 domains displayed in yeast and plant assays, we analyzed *in vitro* DNA binding using biolayer interferometry (Figure 6, Table 1). Analysis of reaction time course data indicated that dissociation of B3-DNA complexes was typically biphasic with distinct fast and slow components (Supplementary Figure S11A). To resolve fast and slow dissociating components of total DNA binding activity, first order off-rate constants *k*_fast_ and *k*_slow_, dissociation time courses were estimated by fitting dissociation time course data to a two-component exponential model using non-linear-least squares (Supplementary Table S6, Supplementary Figure S11A−C). We noted a striking correlation (*R*^2^ = 0.94) between the fast-dissociating fraction of total DNA binding at sub-micromolar protein concentrations and *in vivo* capacity for ABA-independent activation of *CRC* in plants (Supplementary Figure S12A, B). To further resolve the fast- and slow-dissociating DNA binding activities, we fit the full reaction time course data using COPASI (39) to a model with independent low- and high-affinity sites (Supplementary Figure S13). Dissociation constants for low- and high-affinity binding (*K*_d1_ and *K*_d2_, respectively) were then calculated from the rate constants (*K*_d1_ = *k*_fast_/*k*_on1_; *K*_d2_ = *k*_slow_/*k*_on2_; Table 1 and Supplementary Table S6 and S7).

As shown in Figure 6, among the five B3 domains analyzed the binding free energy associated with *K*_d1_, Δ*G*°_1_, was highly correlated (*R*^2^ = 0.99) with ABA-independent activation of *CRC* measured in plants (Figure 3 and 5). In addition, variation in Δ*G*°_1_ aligned qualitatively with differences in activities of B3 domains observed in yeast (Figure 2) though it did not account for a quantitative difference in activities of LEC2-B3 and FUS3-B3. By contrast, the free energy associated with *K*_d2_ correlated weakly (*R*^2^ = 0.27; Supplementary Figure S14) with *in vivo* activity, especially among the four LEC2-B3 and ABI3-B3 related domains, leaving the biological relevance of the tightly bound B3 protein detected by BLI under these conditions unclear. Moreover, the *K*_d1_ estimates for ABI3-B3 and FUS3-B3 are comparable to published *K*_d_ values for these proteins: ABI3-B3, 2.00E−06 M versus 2.4E−06 M (40) and FUS3-B3, 2.63E−07 M versus 1.62E−07 M (9). While we did not find published *K*_d_ values for LEC2-B3 to compare, the *K*_d1_ estimate (3.66E−07 M) was comparable to the *K*_d_ value obtained independently by EMSA analysis (1.98E−07 M; Supplementary Figure S15). FUS3-B3 on the other hand exhibited a qualitatively higher affinity in EMSA experiments (Supplementary Figure S15). The basis for this difference in detection of low-affinity binding activity by EMSA was not determined. Nevertheless, these results overall highlighted the biological relevance of *K*_d1_ for all B3 domains including FUS3-B3.

#### β4-triad effects on DNA binding affinity

The enhanced *in vivo* activity of ABI3-B3 KKS triple mutant relative to ABI3-B3 was associated with a 4-fold decrease in *K*_d1_ corresponding to a favourable 3.44 kJ mol^−1^ decrease in Δ*G*°_1_ (Table 1), whereas *K*_d2_ increased slightly. The reciprocal, negative impact of the LEC2-B3 RRP triple substitution on *in vivo* activity was associated with changes of similar magnitude in *K*_d1_ (2.5-fold increase relative to LEC2-B3) and Δ*G*°_1_ (increased by 2.23 kJ mol^−1^ relative to LEC2-B3). In both cases, the transformations conferred by β4-triad substitutions accounted for greater than half of the total 4.21 kJ difference in Δ*G*°_1_ values of LEC2-B3 and ABI3-B3. Evidently, other amino acid differences account for the remaining ∼30−40% of the parental free energy difference.

### DNA backbone interactions of β4-triad amino acids

To explore the structural implications of the β4-triad in differentiation of LEC2-B3 and ABI3-B3 domains, we analyzed published structures of B3 domains bound to the cognate Sph/RY element (8,9) and constructed homology models of ABI3-B3 using VAL1, LEC2 and FUS3 structures (PDB id: 6FAS and 6J9A, 6J9B, and 6J9C, respectively) (8,9) as templates. In B3-DNA complexes, the β4-strand aligns with the DNA phosphate backbone at an oblique angle with the point of closest approach occurring at β4-triad residue 69. In the LEC2 B3-DNA complex, contact with DNA (3.2 Å separation) is associated with a H-bond between the amino nitrogen of S69 and a backbone phosphate (Supplementary Figure S16A). By contrast, the corresponding nitrogen of proline 69 in VAL1-B3, FUS3-B3 and ABI3-B3 domains lacks capacity for H-bonding (Supplementary Figure S16B–G). In all B3 domain structures (8,9), the positively charged side-chain of residue 66 interacts electrostatically with the DNA backbone. A lysine at position 64 in place of arginine in ABI3-B3 is the sole β4-triad substitution shared by LEC2-B3 and FUS3-B3. The distances of the K64 side-chain from backbone phosphates in LEC2-B3 (6.53 Å) and FUS3-B3 (4.61 Å) DNA complexes are consistent with weak-to-moderate strength electrostatic interaction with DNA.

## DISCUSSION

Our results highlight a dichotomy in the functions of B3 DNA binding domains derived from the architecturally diverse family of transcription factors that recognize the Sph/RY cis-regulatory element during plant embryogenesis. Yeast-one-hybrid and VP1::B3 transgenic plant assays were employed to quantify *in vivo* B3-domain functions in simple and complex architecture contexts, respectively. Remarkably, in both settings' qualitative differences in activities of VAL1-B3, ABI3-B3, LEC2-B3 and FUS3-B3 correlate with the architectural complexity of their parent transcription factors rather than the phylogenetic relatedness of the B3 domain sequences. In the complex architecture setting, VAL1-B3 and ABI3-B3, which are derived from otherwise unrelated multiple-domain transcription factors, support the biologically important function of integrating Sph/RY recognition with ABA hormone signaling mediated by the VP1 COAR domain, whereas in the same context hyper-active LEC2-B3 and FUS3-B3 domains disrupt conditional regulation of *CRC* by partially over-riding the requirement for hormone signaling promoting induction of callus phenotypes in seedlings. Hence, we hypothesize that functional constraints associated with complex protein architecture have shaped evolution of B3 domain activities. However, the B3 domain phylogeny and distinct differences in DNA-binding properties and *in vivo* activities of LEC2-B3 and FUS3-B3 indicate these domains most likely adapted independently to reduced architectures resulting in convergent solutions.

We have identified a clade-specific β4-triad of amino acids implicated in interactions with the DNA backbone as a structural basis for the comparatively recent functional divergence of LEC2-B3 and ABI3-B3 domains within the rosid clade of flowering plants. *In vivo* functional analyses in yeast and plant assays confirm that β4-triad substitutions partially account for divergent activities of LEC2-B3 and ABI3-B3. Remarkably, we find striking correlation between β4-triad effects on DNA binding affinities of B3 domains measured *in vitro* and quantitative differences in *in vivo* activity measured in transgenic plants as well as in yeast.

### The architectural complexity hypothesis

Despite some 400 MY of independent evolution VAL1-B3 and ABI3-B3 domains are more-or-less functionally equivalent in the complex VP1 architecture (Figure 3 and 4, Supplementary Figure S7A). Hence, we propose that VAL1-B3 and ABI3-B3 have evolved and independently maintained similar attenuated activities because in their native environments they are constrained to interoperate with other domains in the protein that interact with DNA and/or chromatin. This coordination of domain functions is essential to the biological roles of VAL1 and ABI3 as physical integrators of chromatin and hormone inputs, respectively. By contrast, while B3 domains of LEC2 and FUS3 are less constrained by intra-protein domain interactions, their biological functions as pioneer activators depend strongly on their autonomous capacities for efficient binding to Sph/RY motifs embedded in a complex genome.

In the developing embryo, an essential biological function of the VP1/ABI3 COAR domain is to promote integration of hormone signaling by localizing ABI3 to promoters that contain an ABA response element (13,14). ABI3 binding would be synergistically strengthened in promoters that also contain the Sph/RY motif recognized by the B3 domain. In that environment, an attenuated B3 DNA binding affinity is likely required to ensure an optimal conditional interaction of B3 and COAR activities. Conversely, endowing the B3 domain with a high, autonomous DNA binding affinity would disrupt the combinatorial logic of hormone signaling by promoting Sph/RY recognition independent of ABA-regulated COAR activity—i.e. the effect conferred by VP1::LEC2-B3, VP1::FUS3-B3 and VP1::ABI3-B3 KKS transgenes. We suggest that chromatin binding domains of VAL transcription factors have an analogous role in localization of VAL1-B3 to chromatin domains (3). Hence, independent selection for attenuated DNA binding affinity has likely preserved the biologically important integrative functions of ABI3 and VAL1. Consistent with that hypothesis we find a strong correlation between DNA binding affinity and ABA-independent (autonomous) activities of B3 domains in plants (Figure 6).

### The β4-triad structural basis for LEC2-B3 and ABI3-B3 functional differences

The close phylogenetic relationship and comparatively recent evolutionary divergence of ABI3-B3 and LEC2-B3, derived from transcription factors with complex and reduced architectures respectively, facilitated identification of a clade-specific triad of amino acid substitutions in the β4-strand of the B3 domain as a putative structural basis for functional differentiation. We confirmed that hypothesis by showing that reciprocal β4-triad substitutions were sufficient to partially interconvert *in vivo* activities of LEC2-B3 and ABI3-B3 domains in both yeast (Figure 2) and transgenic plants (Figure 5).

### Correlation of DNA binding affinity with *in vivo* activity

We find a striking correlation of *in vitro* DNA binding affinity (expressed as Δ*G*° of DNA binding) with differences in the *in vivo* activities of B3 domains (Figure 6). This quantitative linear relationship between the binding free energy (Δ*G*°_1_) and *in vivo* hormone-independent *CRC* activation measured in transgenic plants also accounts qualitatively for relative activities of B3 domains in yeast. The correlation between plant and yeast assays is not precise, FUS3-B3 has a slightly lower *K*_d1_ than LEC2-B3 but is less active in yeast. Overall, the reciprocal effects of β4-triad substitutions on Δ*G*°_1_ account for greater than half of the binding free energy difference between parental LEC2-B3 and ABI3-B3 domains. The incomplete transformation is consistent with the partial interconversion of B3 domain activities measured *in vivo*. Not surprisingly, other amino acid differences (e.g. the clade-specific L77V substitution, Figure 1B) presumably contribute to functional differentiation of LEC2-B3 and ABI3-B3 domains.

Importantly, our results indicate that Δ*G*°_1_ is an extraordinarily accurate predictor of *in vivo* activity in plants (Figure 6). We anticipate that this relationship can be further tested and extended by *in vitro* analysis of LEC2-B3 P and ABI3-B3 S substitutions that are predicted to have intermediate Δ*G*°_1_ values proportional to their *in vivo* activities. Hence, there is more to be gleaned from analysis of structural differences that distinguish ABI3-B3 and LEC2-B3.

### β4-triad interactions with the DNA backbone

The β4-triad amino acid substitutions are implicated in interactions with the DNA backbone that evidently alter DNA binding affinity while preserving base-specificity. Notably, β4-triad amino acids are not directly implicated in base contacts that determine sequence specificity of B3 domain binding to DNA (Supplementary Figure S16). Among the β4-triad variants, the serine-proline substitution at position 69 has the largest effect. The S69P mutation in LEC2-B3 P is sufficient to abolish activity in yeast (Figure 2) though it retains partial ABA-independent activity in plants (Supplementary Figure S9B). Conversely, P69S partially activates ABI3-B3 S in both yeast and plant assays (Figure 2C and Supplementary Figure S9). We attribute the impact of the S69P substitution to loss of capacity for H-bonding between the peptide amino N and a phosphate of DNA (Supplementary Figure S16A). Interestingly, the 4.21 kJ mol^−1^ difference in Δ*G*°_1_ values of ABI3-B3 and LEC2-B3 is comparable to the energy of a single H-bond. However, functional analyses indicate that the S69P substitution accounts for only part of this difference. Hence, other subtle shifts in H-bond networks and charge distribution near the DNA backbone likely contribute to differences in DNA binding affinity. In the plant assay, the pair of lysine-arginine substitutions at positions 64 and 66 in LEC2-B3 RRP and ABI3-B3 KKS induce roughly reciprocal ∼1 log_10_ unit changes in ABA-independent *CRC* expression relative to the respective LEC2-B3 P and ABI3-B3 S single mutants (Supplementary Table S4). By that measure, their combined impact is on a par with the impact of the serine-proline substitution at position 69. While the lysine-arginine substitutions conserve positive charge, models of ABI3-B3 and ABI3-B3 K64 mutant domains suggest that differences in side-chain length and H-bonding capacity alter distance of the charge from the DNA backbone (compare Supplementary Figure S16F with models of wildtype ABI3 in parts D, E and G). Consistent with this pattern, FUS3-B3, which has comparatively high DNA binding affinity, has β4-triad lysine K64 in common with LEC2-B3. In structures of diverse B3 domains bound to their specific targets, amino acid 64 interacts with a ‘clamp phosphate’ located at the boundary of the target site that is thought to promote a protein orientation conducive to base-specific contacts (41).

### Independent adaption of LEC2-B3 and FUS3-B3 to reduced architectures

Our phylogenetic and functional analyses indicate that LEC2-B3 and FUS3-B3 have independently adapted to reduced architectures—evidently arriving at different structural solutions. Although both have evolved high DNA binding affinity, the domains likely have evolved under somewhat different constraints. Non-redundant functions of LEC2 and FUS3 in embryogenesis are differentiated in part by their temporal order of expression (5,23). High affinity binding is likely crucial to the biological role of LEC2 as a pioneer activator of Sph/RY containing genes during early embryogenesis (17,18,21–23). Peak expression of FUS3 occurs several days later in embryogenesis (18,20,21,23,42,43) under a modified landscape of open chromatin already established by LEC2 and LEC1. In that environment, a high affinity of FUS3-B3 for Sph/RY may be essential for maintaining the embryogenic state (5). ABA-independent activities of VP1::FUS3-B3 (Figure 3F) evidently coincide with ectopic activation of LEC1 in leaves promoting frequent callus induction (Figure 4). Consistent with the evidence that FUS3-B3 and LEC2-B3 independently acquired higher DNA binding affinities after separation from ABI3-B3 ancestors, they have only one β4-triad residue in common (K64). Other amino acid changes that account for divergence of FUS3-B3 and ABI3-B3 remain to be identified. Interestingly, FUS3-B3 has a clade-specific arginine substitution in place of glycine at position 26 (Figure 1B). In the FUS3-B3 structure (9) the R26 side-chain extends toward the DNA backbone forming an electrostatic interaction that is unique to FUS3-B3.

### DNA binding domain functional adaptations that preserve DNA sequence specificity

Our results highlight structural variation affecting non-specific electrostatic protein-DNA interactions as a general mechanism enabling adaptation of DNA binding affinity while preserving DNA sequence specificity. Because a non-sequence-specific electrostatic contribution to the total binding free energy is a universal feature of protein-DNA interactions (44,45), this mechanism has broad relevance to understanding functional diversification of transcription factor families. Because architectural diversification is a common mechanism of innovation in transcription factor networks that underly plant and animal development, we suggest that architectural constraints are a key driver for functional differentiation of conserved DNA binding domains.

## DATA AVAILABILITY

Sequence data from this article can be found in the Arabidopsis Information Resource (TAIR) and GenBank/EMBL databases under the following accession numbers: ABI3, TAIR AT3G24650; FUS3, TAIR AT3G26790; LEC2, TAIR AT1G28300; VAL1, TAIR AT2G30470; VAL2, TAIR AT4G32010; VAL3, TAIR AT4G21550; LEC1, TAIR AT1G21970; CRC, TAIR AT4G28520; TUB2, TAIR AT5G62690; and VP1, GenBank NM_001112070.

## Supplementary Material

gkab257_Supplemental_File

## Notes

Present address: Haiyan Jia, Department of Biology, University of North Carolina, Chapel Hill, NC 27599-3280, USA.
