# Supplementary material for: Structural variation affecting DNA backbone interactions underlies adaptation of B3 DNA binding domains to constraints imposed by protein architecture
Source: Nucleic Acids Res. 2021 Apr 19;49(9):4989–5002. doi: 10.1093/nar/gkab257 (PMC8136769; doi:10.1093/nar/gkab257)
Supplement: gkab257_Supplemental_File [file gkab257_supplemental_file.pdf]

Supplemental methods

Gel mobility shift assay

In the gel mobility shift assay, DNA binding reactions contained 7.5 fmol of Sph2-probe and various concentration of proteins suspended in a 15ul of binding reactions (LightShift Chemiluminescent EMSA kit, Catolog # 20148, Thermo Scientific). The components in the reactions [1X Binding buffer, 50ng/ul Poly (dI.dC), 2.5% of Glycerol, 5mM $MgCl_2$, 0.05% NP-40, and 0.5 nM DNA probe]. The reactions were incubated at room temperature for 20 min and then resolved on 6% polyacrylamide gel run in 0.5 X TBE buffer at 4 ºC. The remaining steps were performed as described in the LightShift Chemiluminescent EMSA kit (Thermo Scientific). The images were captured by a CCD camera or X-ray film. Images were analyzed by densitometry using image J.

Chromatin immunoprecipitation-quantitative PCR (ChIP-qPCR) assays

ChIP assays were performed as the protocol described by Komar et al., (1). Briefly, 1.5 g Arabidopsis leaf tissue was fixed with 1% formaldehyde under vacuum and was grinded with liquid nitrogen. After nuclei were extracted and lysed, chromatin was fragmented to most abundant size at ~500 bp by Bioruptor 300 (Diagenode) for 30 cycles with the settings at high 30 sec ON/30 sec OFF at 4°C. The supernatant was pre-cleaned by incubation with Protein A/G MagBeads (Cat. No. L0027, Genscript) at 4 °C for 1 hour, and then immuno-precipitated by anti-GFP antibody (Cat. No, 50430-2-AP, Proteintech) coated protein A/G magnetic beads at 4 °C overnight. A mock sample without any antibody was prepared simultaneously. After reverse-crosslink, the precipitated DNA was purified and used as the templates for qRT-PCR with Luna Universal qPCR master mix (M3003G). The primer sequences were shown in Supplementary Table S3.

References

1. Komar, D.N., Mouriz, A., Jarillo, J.A. and Pineiro, M. (2016) Chromatin Immunoprecipitation Assay for the Identification of Arabidopsis Protein-DNA Interactions In Vivo. J. Vis. Exp., e53422.

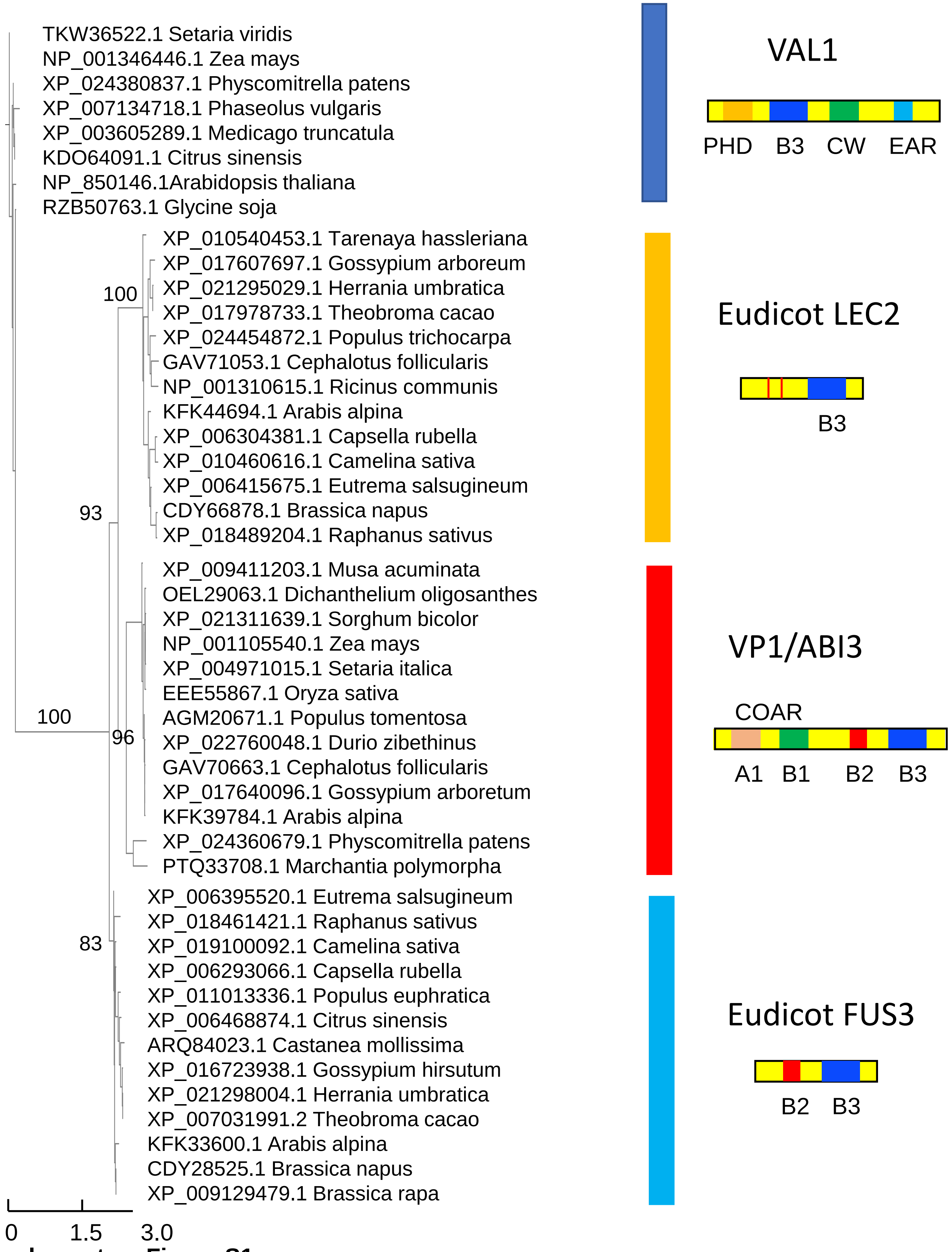

Supplementary Figure S1

Phylogenetic tree of AFL and VAL B3 domain sequences. B3 domain sequences (120 amino acids) were aligned using MAFFT and a maximum likelihood tree constructed in PhyML with bootstrap support based on BOOSTER. Domain architectures are diagrammed for each of the four principle B3 clades, VAL1, dark blue rectangle, LEC2, gold rectangle, VP1/ABI3 red rectangle, FUS3, light blue rectangle.

A triad of amino acids in β4 differentiate LEC2 and ABI3:

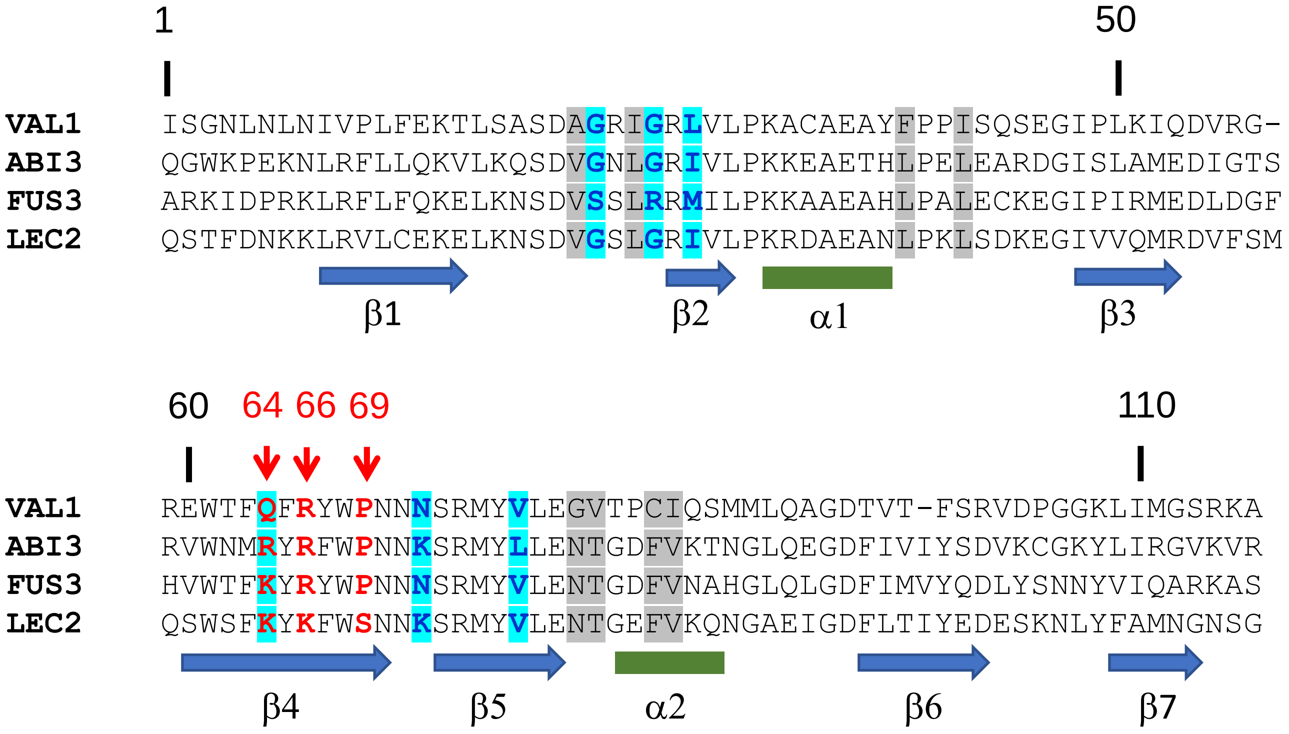

Supplementary Figure S2. Alignment of Arabidopsis AFL and VAL1 B3 domains. Clade-specific variant positions are highlighted as described in Figure 1B. b4-triad positions are colored red and indicated by arrows. Beta-sheet (block arrows) and helix regions (rectangles) are based on a consensus of B3 structures. Q64, R66, and P69 in VAL1-B3 domain are Q345, R347, and P350 in VAL1 full-length protein; R64, R66, and P69 in ABI3-B3 domain are R623, R625, and P628 in ABI3 full-length protein; K64, R66, and P69 in FUS3-B3 domain are K143, R145, and P148 in FUS3 full-length protein; and K64, K66, and S69 in LEC2-B3 domain are K222, K224, and S227 in LEC2 full-length protein.

Reporter

>Sph2: GATCATGCATGGACGACACGGATCATGCATGGACGACACG

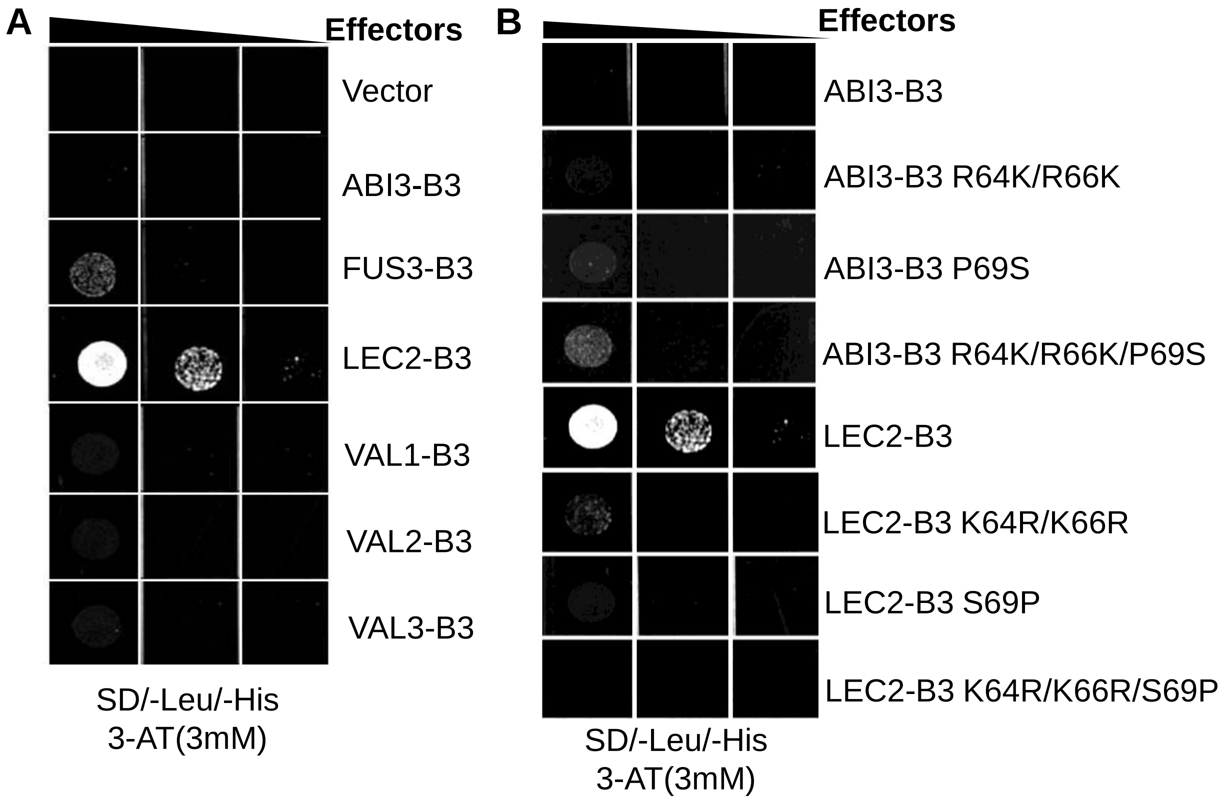

Supplementary Figure S3

Functional analysis of B3 domains in a Y1H assay showing partial interconversion of LEC2 and ABI3 B3 domain activities. AFL and VAL B3 domain sequences used in the effector constructs are shown in Supplemental Figure S2. The Y1H system: yeast reporter strains carry the *HIS3* gene under control of a *Sph2* minimal promoter. The core Sph motifs are underlined in the Sph2 minimal reporter sequence. All Y1H effectors were fusions to a GAL4 activation domain in the pGADT7 vector, which contains a *LEU2* marker for selection in yeast. (**A, B**), Colony growth assays of yeast transformed B3 domain effectors from either AFL or VAL. Equal volumes of ten-fold serial dilutions of effector transformed yeast cells were spotted on SD/-Leu/-His plus 3mM 3-AT media, and incubated at 30°C for 4 days. (**A**) AFL and VAL WT B3 domain effectors; (**B**) LEC2 B3 and ABI3 B3 domain mutant effectors.

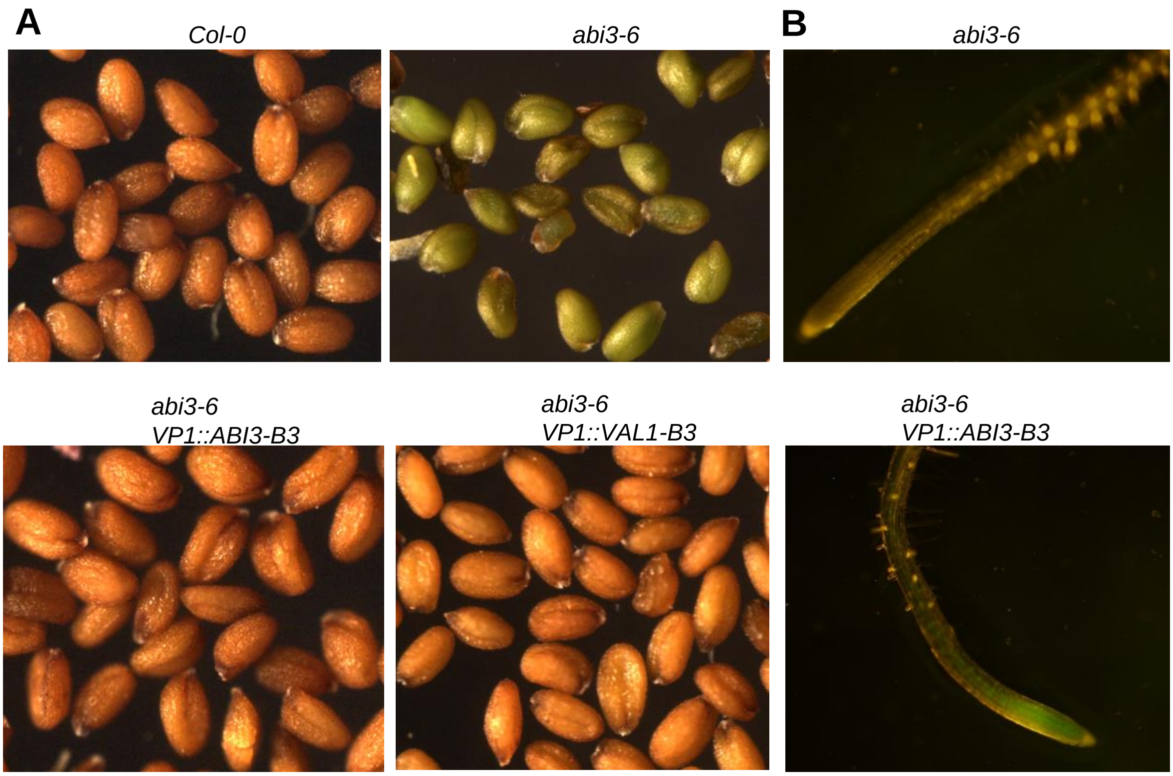

Supplementary Figure S4

The seed phenotype and GFP expression of the *Pro35S:VP1::B3* transgenic plants. (**A**) *Pro35S:VP1::ABI3-B3* and *Pro35S:VP1::VAL1-B3* complemented *abi3-6* mutant seed green color and desiccation intolerance phenotypes. Mature siliques were collected from Columbia (*Col-0*) wild type, *abi3-6*, *abi3-6 Pro35S:VP1::ABI3-B3* and from *abi3-6 Pro35S:VP1::VAL1-B3* T3 homozygous transgenic plants. (**B**) GFP expression was observed in root tips of 12-d-old *abi3-6 Pro35S:VP1::ABI3-B3* T3 homozygous transgenic plants

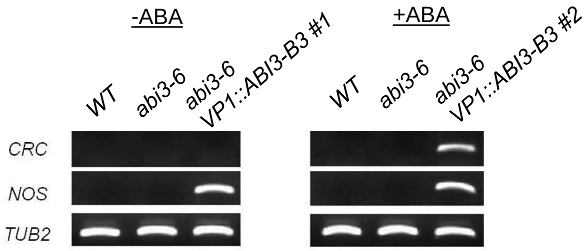

Supplementary Figure S5

CRC expression in the leaf tissue of *WT*, *abi3-6*, and the T1 line of *abi3-6 Pro35S:VP1::ABI3-B3* with and without ABA treatment. RNA was isolated from leaves of 14-d-old T1 transgenic seedlings in absence or in presence of ABA treatment. For this experiment, 2 independent T1 *abi3-6 Pro35S:VP1::ABI3-B3* seedlings that had normal phenotypes were used. RT-PCR was performed to detect the transcript of downstream target *CRC* and transgene. Primers targeted to the NOS-terminator region were used for quantify the transcript level of the *VP1::ABI3-B3* transgene. The amount of total RNA used for each reaction is 25ng. *TUB2* was used as an endogenous control.

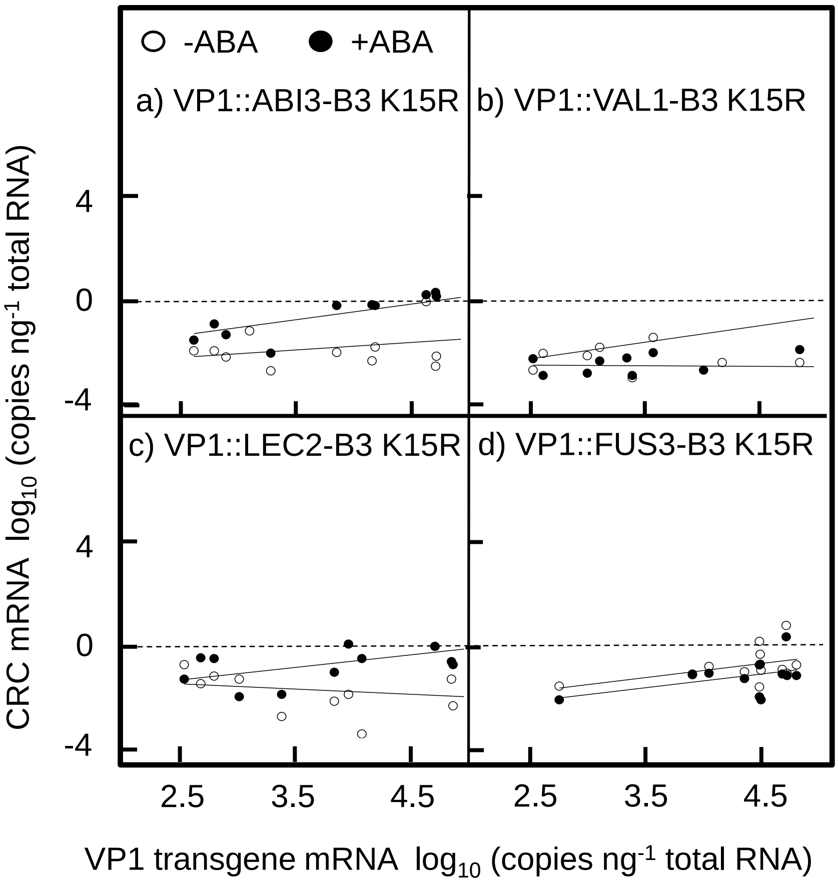

Supplementary Figure S6

The K15R loss of function mutant abolishes *in vivo* activities of ABI3, LEC2, FUS3, and VAL1 B3 domains. Expression of the indicated *VP1::B3* transgene and *CRC* was quantified in individual transformed T1 seedlings by qPCR with and without 24 h treatment with 5 µM ABA.

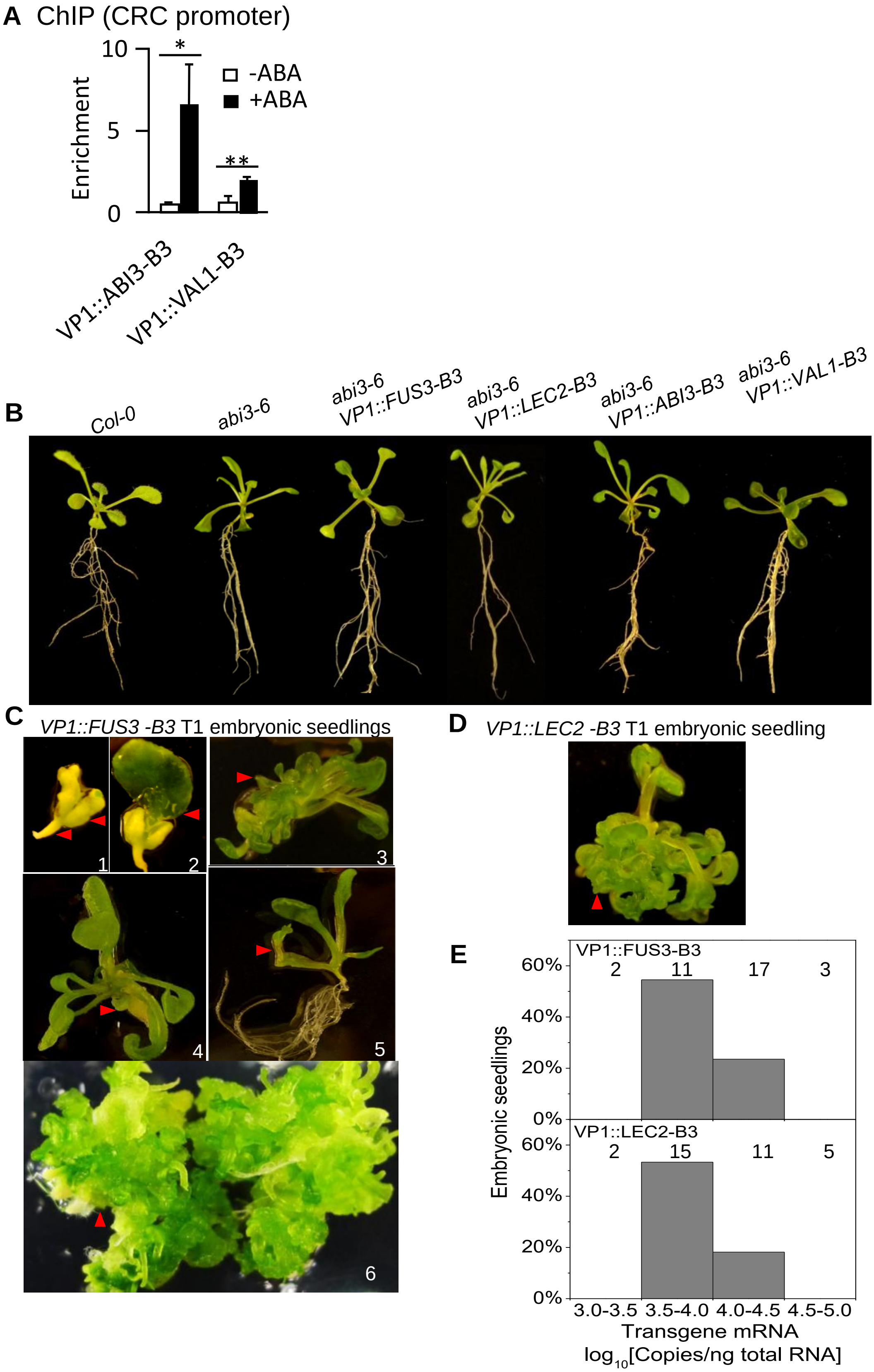

Supplementary Figure S7

Supplementary Figure S7

ChIP and phenotype of transgenic plants. (**A**) ChIP confirmation of ABA dependent binding of VP1::ABI3-B3 and VP1::VAL1-B3 to the *CRC* promoter in Arabidopsis plants. (B) T1 transgenic seedlings with normal phenotypes. T1 transformants that complemented the desiccation intolerant seed phenotype of *abi3-6* were grown 14-d on MS media containing Hygromycin. (**B**).Occurrence of embryonic callus formation in *P35S:VP1::LEC2-B3* and *P35S:VP1::FUS3-B3* transformants. (**C**) The *abi3-6 Pro35S:VP1::FUS3-B3* T1 transgenic seedlings having a variety of embryonic seedling phenotypes involving callus formation (red arrows). 1, a seedling with arrested embryos; 2, a seedling with arrested embryos that produced a shoot from the SAM; 3, a seedling with many adventitious shoots but no primary root; 4, a seedling with near-normal shoot development, but callus formation in the root region; 5, a seedling with callus in the cotyledon region; 6, an embryonic seedling with extensive callus tissue as well as adventitious shoot and root formation after one month culture on MS media. (**D**) Representative *abi3-6 Pro35S:VP1::LEC2-B3* T1 transgenic seedling that have embryonic seedling phenotype. (**E**) Histogram of the frequency distribution of embryonic seedlings. Frequency distribution of embryonic seedlings classified based on transgene expression in *abi3-6 Pro35S:VP1::FUS3-B3* and *abi3-6 Pro35S:VP1::LEC2-B3* transgenic lines. The total number of seedlings in each class is indicated above the bar.

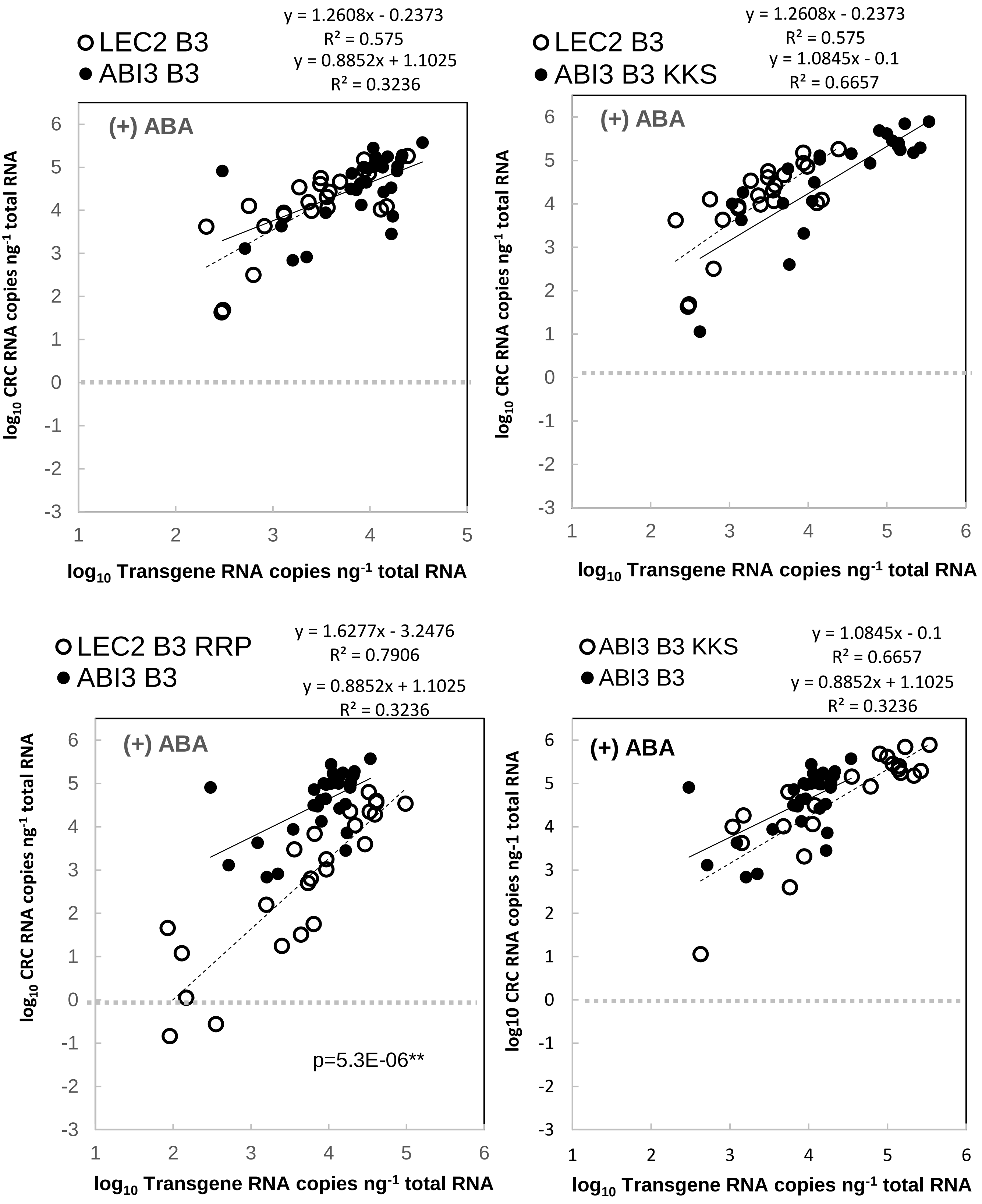

Supplementary Figure S8

Effects of β4-triad substitutions on ABA-dependent activities of VP1::ABI3-B3 and VP1::LEC2-B3. Transgenic seedling were treated with 5 μM ABA. Regression lines for open symbols, dashed; regression lines for closed symbols, solid. Horizontal dashed lines at 0 are included for reference.

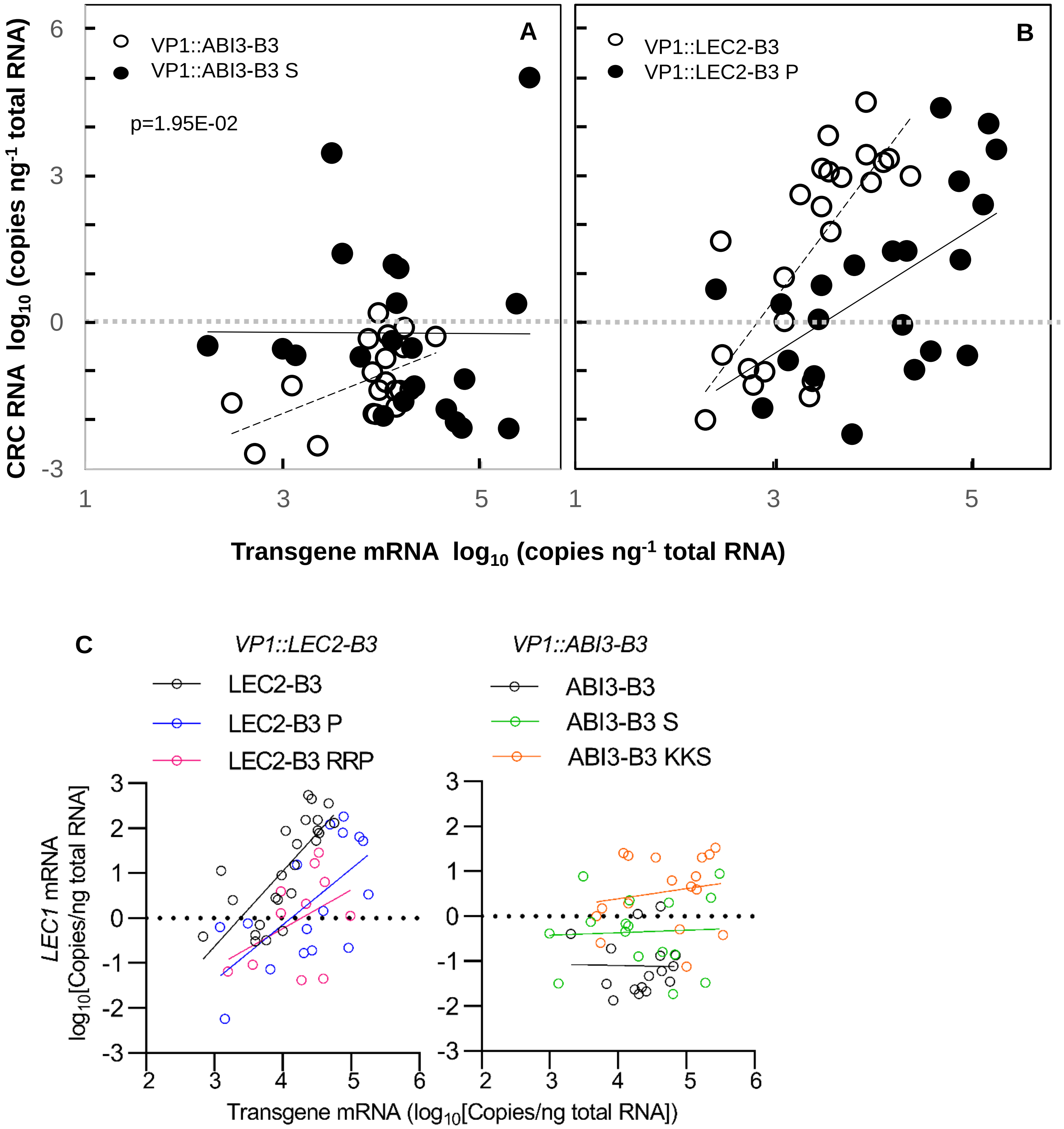

Supplementary Figure S9

(A, B) Effects of S69P and P69S substitutions on ABA-independent activities of VP1::ABI3-B3 and VP1::LEC2-B3. **(C)** *LEC1* expression in the *P35S:VP1::LEC2-B3* and *P35S:VP1::ABI3-B3* transformants. 14-d-old T1 *Pro35S:VP1::B3* transgenic seedlings were analyzed for transgene and *LEC1* expression using qPCR. Total RNA was isolated from leaf tissue of normal seedlings.

>VP1-B3
DIHHRLAAASDKRQGAKADKNLRFLLQKVLKQSDVGSLGRIVLPKKEAEVHLPELKTRDGISIPMEDIGT
SRVWNMRYRFWPNNKSRMYLLENTGEFVRSNELQEGDFIVIYSDVKSGKYLIRGVKVRPPPAQEQGSGSS
>FUS3-B3
DIHHRLAAASDKRARKIDPRKLRFLFQKELKNSDVSSLRRMILPKKAAEAHLPALECKEGIPIRMEDLDG
FHVWTFKYRYWPNNNSRMYVLENTGDFVNAHGLQLGDFIMVYQDLYSNNYVIQARKASEPPAQEQGSGSS
>LEC2-B3
DIHHRLAAASDKRQSTFDNKKLRVLCEKELKNSDVGSLGRIVLPKRDAEANLPKLSDKEGIVVQMRDVFS
MQSWSFKYKFWSNNKSRMYVLENTGEFVKQNGAEIGDFLTIYEDESKNLYFAMNGNSGKQNEPPAQEQGS
GSS
>ABI3-B3
DIHHRLAAASDKRQGWKPEKNLRFLLQKVLKQSDVGNLGRIVLPKKEAETHLPELEARDGISLAMEDIGT
SRVWNMRYRFWPNNKSRMYLLENTGDFVKTNGLQEGDFIVIYSDVKCGKYLIRGVKVRQPPAQEQGSGSS
>VAL1-B3
DIHHRLAAASDKRQISGNLNLNIVPLFEKTLSASDAGRIGRLVLPKACAEAYFPPISQSEGIPLKIQDVR
GREWTFQFRYWPNNNSRMYVLEGVTPCIQSMMLQAGDTVTFSRVDPGGKLIMGSRKAANPPAQEQGSGSS

Supplementary Figure S10

Amino acid sequences of B3 domains of GST-B3 recombinant proteins used in the *in vitro* binding assays. VP1-B3 sequence contains 140 amino acid residues was used previously in in vitro DNA binding analysis. The AFL and VAL B3 domain residues in black font were used in Y1H and also used for making all the domain replacement in the *VP1::B3* chimeral transgenes. The blue residues of N and C terminal of VP1 B3 domain are contained in all the AFL and VAL1 B3 domains tested in the *in vitro* binding assays.

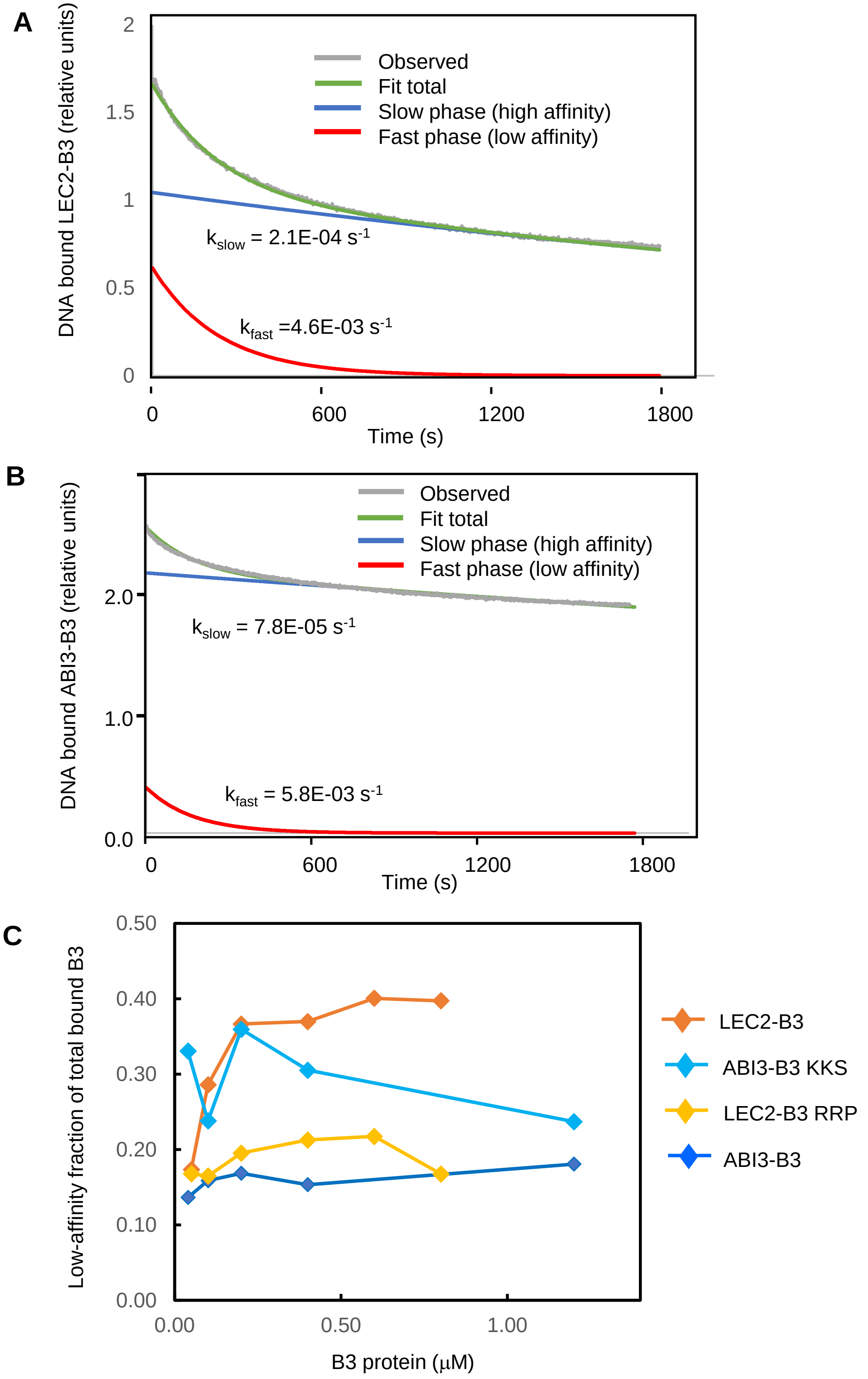

Supplementary Figure S11 (**A-B**) Deconvolution of fast (red) and slow (blue) phases of LEC2-B3/DNA and ABI3-B3/DNA complex dissociation. Dissociation time course data were fit to a two-component exponential model by non-linear least square in R (R-project.org). Time courses of 0.4 μM protein reactions are shown. The k_{fast} and k_{slow} estimates used for kinetic modeling in COPASI (Supplementary Table S6) were the means from time courses of three protein concentrations. (**C**) Protein concentration dependence of low-affinity binding. Estimate standard errors were less than symbol size.

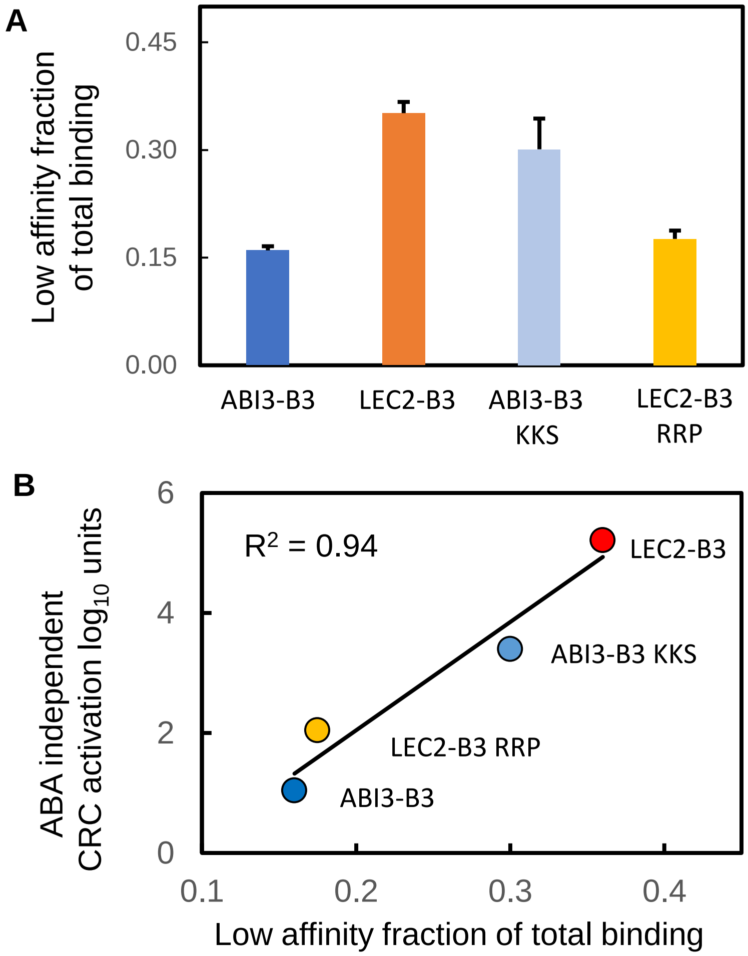

Supplementary Figure S12. Correlation of low-affinity binding with *in vivo* activity in transgenic Arabidopsis. Dissociation time course data were fit to a two-phase exponential model by non-linear least squares (R-project.org). (**A**) Mean values of parameter b from 3 sub-micromolar protein concentrations (Supplementary Table S6). (**B**) Correlation of values in (**A**) with ABA-independent *CRC* activation measured in Arabidopsis (adapted from Figure 5G, red bars).

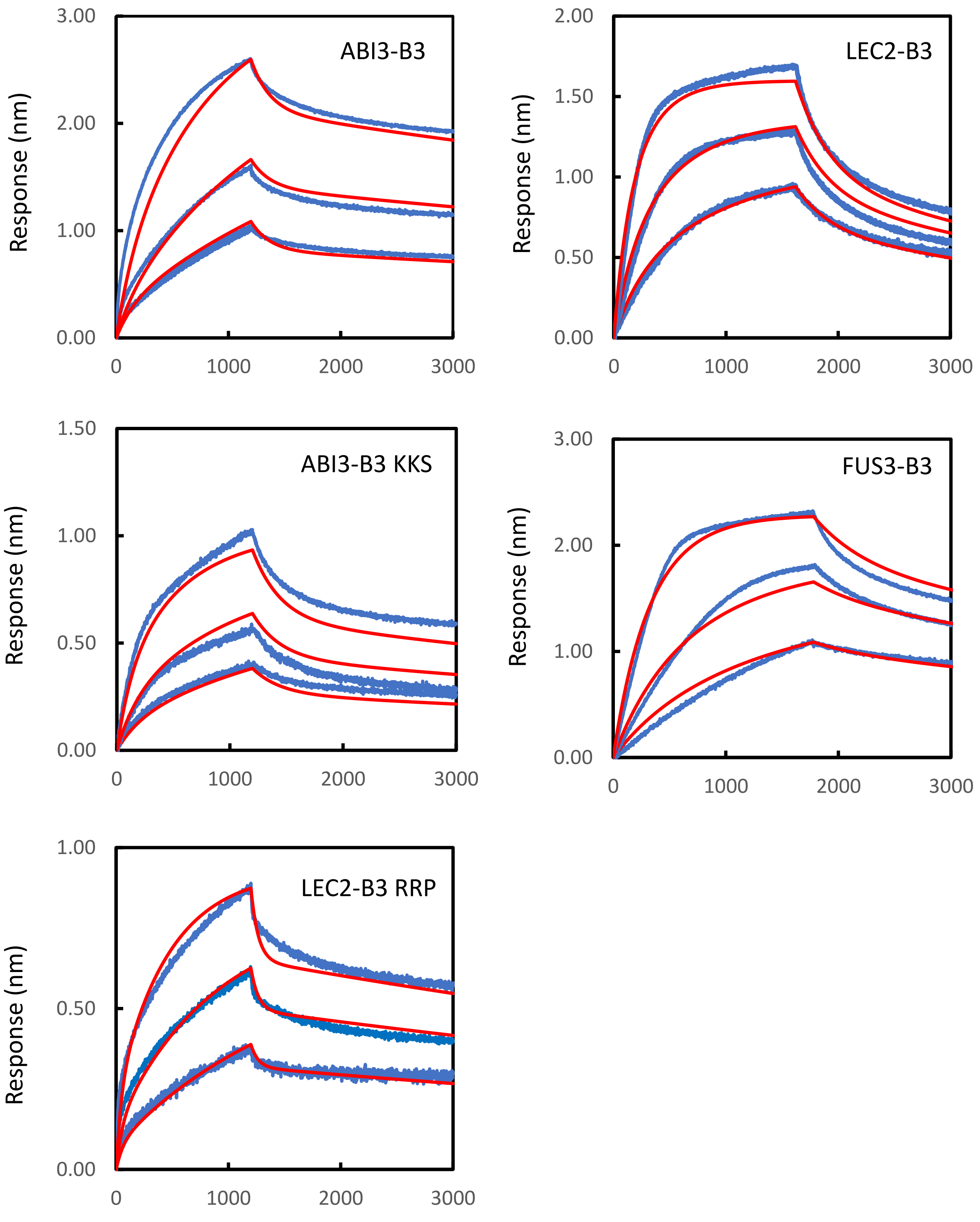

Supplementary Figure S13A. B3 time course profiles fit to a two-site model. Time course data were fit to the model including independent low and high affinity sites using the Parameter Estimation tool of COPASI using k_{fast} and k_{slow} estimates in (Supplementary Table S6). Time courses for three protein concentrations were fit simultaneously. Protein concentrations of 0.1, 0.2 and 0.4 µM were fit for ABI3-B3, LEC2-B3, ABI3-B3 KKS and LEC2-B3 RRP. For FUS3-B3 0.02, 0.04 and 0.1 µM protein reactions were fit. Blue, observed values; red, predicted.

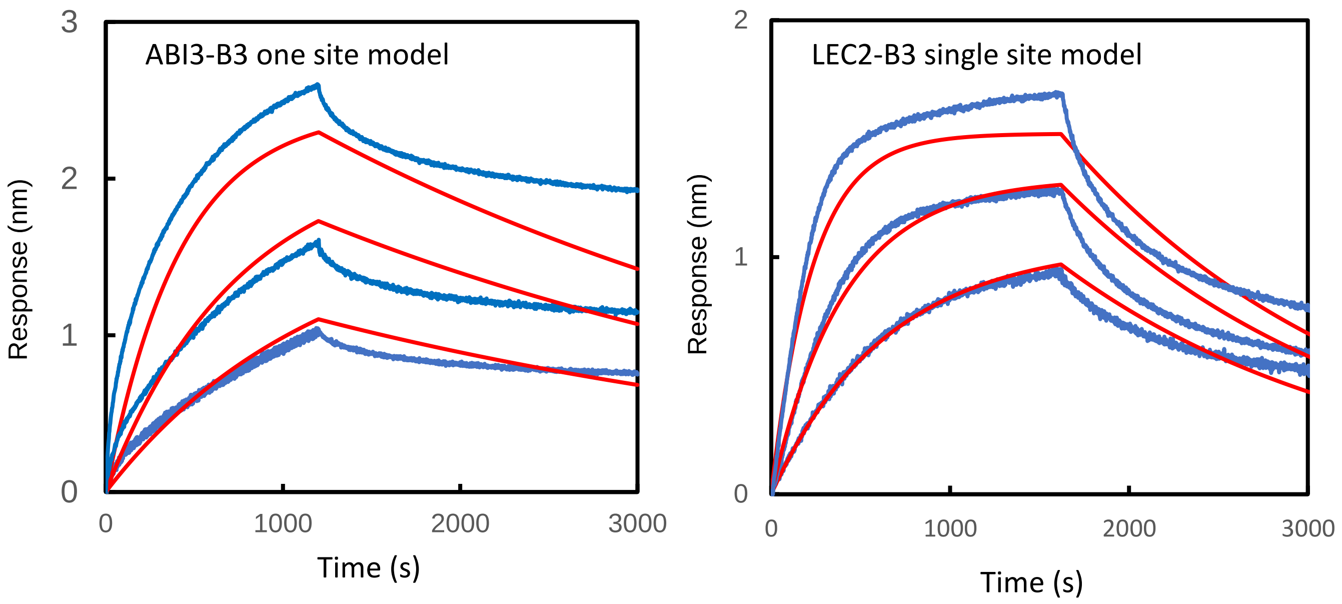

Supplementary Figure S13B. B3 time course profiles fit to single-site model. Time course data were fit to model for a single site using the Parameter Estimation tool of COPASI. Protein concentrations of 0.1, 0.2 and 0.4 μM were fit for ABI3-B3 and LEC2-B3. Blue, observed values; red, predicted.

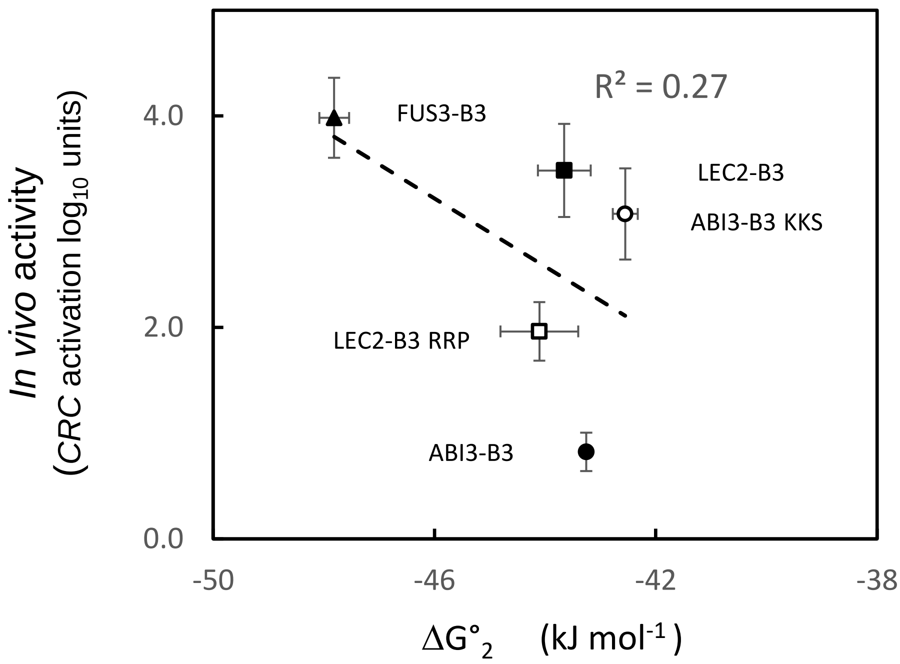

Supplementary Figure S14. Relationship between high-affinity DNA binding and *in vivo* activity. The binding free energy associated with the high affinity site (K_{d2}) detected in vitro is plotted on the x-axis. The y-axis is ABA-independent activation of *CRC* measured in transgenic Arabidopsis. Gene expression is expressed in log_{10} units above a base line of 0.01 *CRC* mRNA copies per ng total RNA. R^2 was determined by linear regression (dashed line). Error bars indicate standard errors of the mean for gene expression (vertical) and standard errors for free energy estimates (horizontal).

Sph2 probe: 5' biotin-CACGGATCATGCATGGACGACACGGATCATGCATGGACGACACG-3'

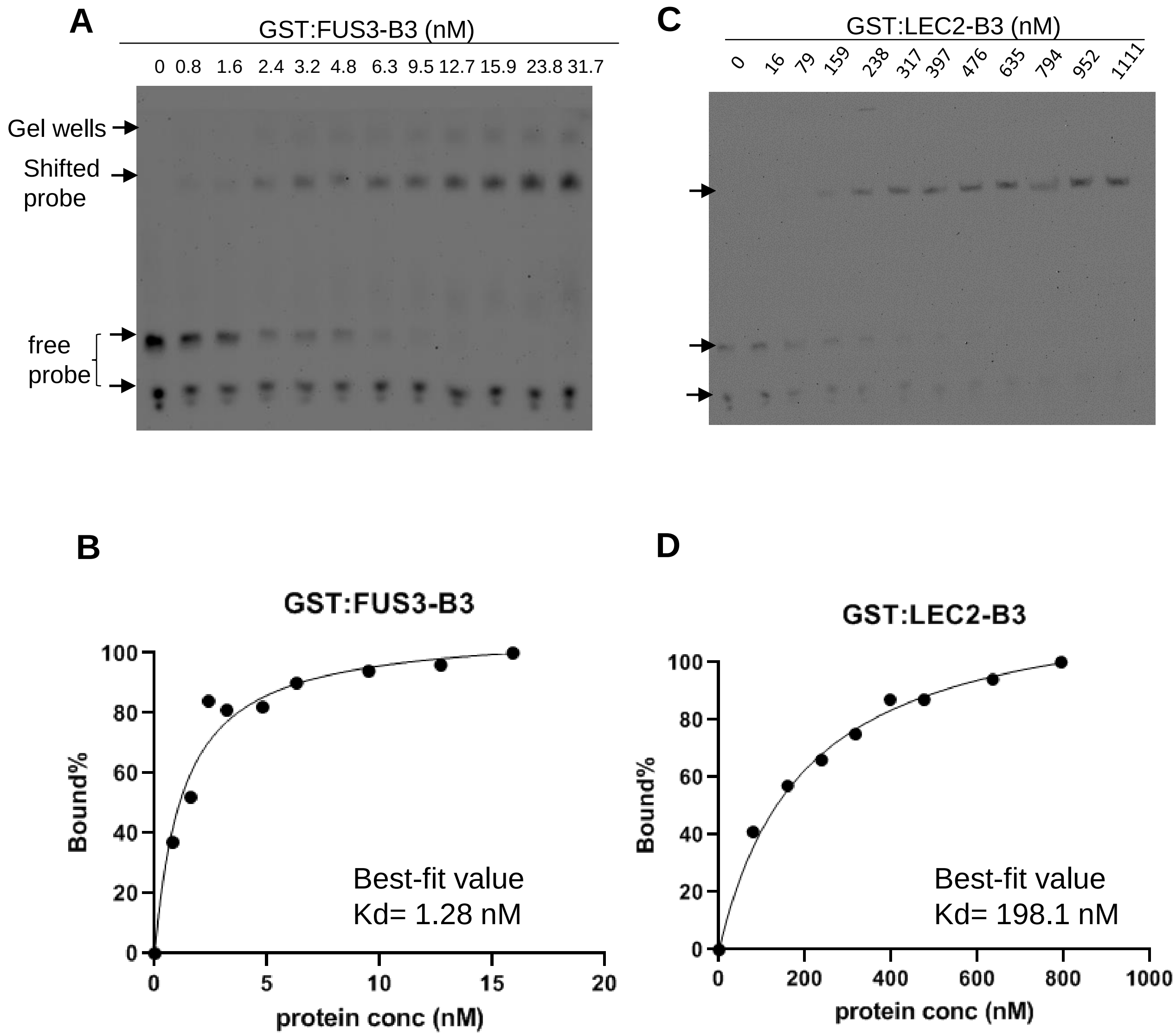

Supplementary Figure S15

Analysis of B3 domain DNA binding activity by gel mobility shift assay. GST:B3 binding to the Sph2 DNA probe was determined by gel mobility shift assays varying protein concentration. The probe sequence is shown at the top of the figure. DNA probe concentration is 0.5 nM. Chemiluminescence images in (**A**) and (**C**) were captured by a CCD camera. Free probe forms two bands, the upper band is the dsDNA, and the lower band is ssDNA. The fraction of the total probe shifted was quantified by densitometry using image J. Binding curves GST:FUS3-B3 (**B**) and GST:LEC2-B3 (**D**) were determined by nonlinear least squares fitting the hyperbolic binding equation: Y = Bmax*X/(Kd + X), where Y is the percentage of total probe shifted, X is the protein concentration in nM units, and K_d is the dissociation constant (in nM units). GraphPad Prism 8.4 software was used to fit the data and make the graphs.

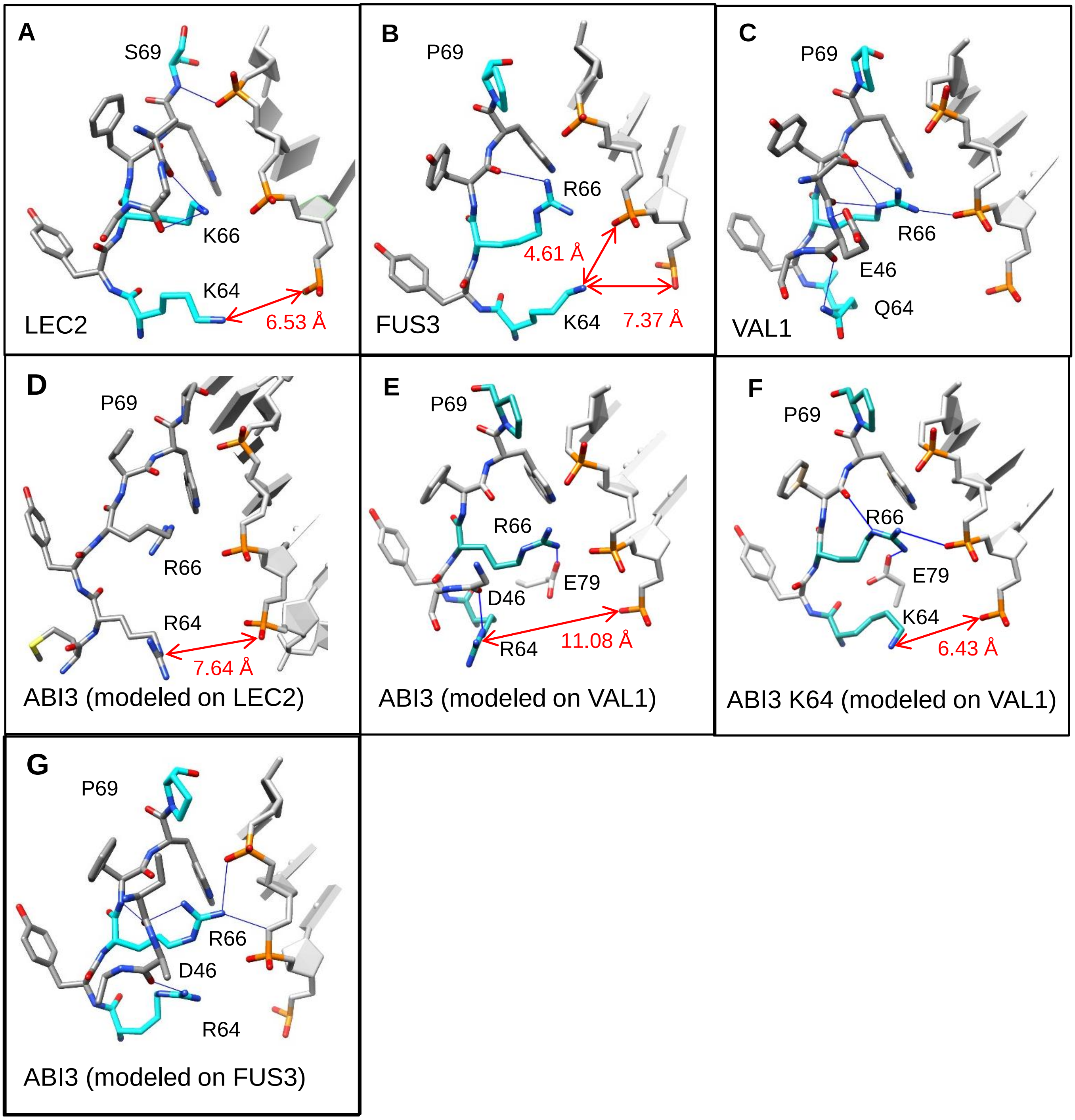

Supplementary Figure S16

(**A**) Peptide backbone nitrogen of β4-triad residue S69 forms a H-bond with a DNA backbone phosphate that is not present in FUS3-B3 (**B**) and VAL1-B3 (**C**). H-bonding of R64 with the backbone carbonyl of D46 in independent ABI3 models (**D, E, G**). (**F**) ABI3-B3 R64K substitution modeled on VAL1-B3. ABI3 models based on VAL1-B3 structure (6FAS.pdb), FUS3-B3 (6J9B.pdb) and LEC2-B3 (6J9C.pdb). H-bonds (blue lines) were identified using the findHBond tool in Chimera with relaxed constraints. Red arrow, distance from R64 guanidium or K64 side-chain nitrogen to nearest phosphate.

Supplementary Table S1. Primers used in Y1H study

Target region	Gene	Sequences: 5'>3' (ClaI: ATCGAT; SacI: GAGCTC)
B3 domain	ABI3	GGCATCGATACCAGGGATGGAAACCAGAAAA
		GTAGAGCTCGTTGTCTTACTTTAACCCCTC
	FUS3	GGCATCGATACGCACGTAAAATTGACCCAAG
		GTAGAGCTCGTTCCGATGCTTTTCTTGCTT
	LEC2	GGCATCGATACCAGTCCACATTTGATAACAAG
		GTAGAGCTCGTTCATTTTGTTTTCCCGAAT
	VAL1	GGCATCGATACCAGATCTCTGGAAATTTGAA
		GTAGAGCTCGATTAGCTGCCTTCCTGGAAC
	VAL2	GGCATCGATACCAGTATCCTCATCTCTCAA
		GTAGAGCTCGGTTCGTCGCTTTACGGTATC
	VAL3	GGCATCGATACCAATCAAAAGAATCTGAATC
		GTAGAGCTCGAGTAATCGAAGCCTTTCTGA
AFL B3 mutants	ABI3 R64K/R66K	GTGTTTGGAACATGAAATACAAGTTTTGGCCTAAC
	ABI3 P69S	CGCTACAGGTTTTGGTCCAACAACAAAAGCAGG
	ABI3 R64K/R66K/P69S	GTGTTTGGAACATGAAATACAAGTTTTGGTCCAA CAACAAAAGCA
	LEC2 K64R/K66R	GTCTTGGTCTTTCCGCTACAGGTTTTGGTCCAAT
	LEC2 S69P	ATACAAGTTTTGGCCTAATAACAAGAGCAG
	LEC2 K64R/K66R/S69P	GTCTTGGTCTTTCCGCTACAGGTTTTGGCCTAAT AACAAGAGCAG

Supplementary Table S2.

Primers used in transgene construction and transgenic line analysis

Purpose	Targets	Primer	Sequences: 5'>3' (*EcoRV*: GATATC; *SacI*: GAGCTC; *XbaI*; TCTAGA; *XhoI*: CTCGAG)
Cloning full length *VP1* cDNA into TOPO vector, *XbaI/XhoI* sites were added to 5' and 3' end.	*VP1*	VP1-*XbaI*	AGCGTCTAGAATGGAAGCCTCCTCCGGCTCGTCGC
		VP1-*XhoI*	GTCGCTCGAGGATGCTCACCGCCATCTGGCTCATG
Creat *EcoRV* and *SacI* sites at VP1 B3 domain borders by mutagenesis PCR.	*VP1*	VP1-*EcoRV*	CGCAGCGGCTTCAGATGATATCCAGGGCGCCAAGGCGG
		VP1-*SacI*	CGTGAAGGTAAGGCCCGAGCTCGCGCAAGAGCAAGGCAG
Cloning the B3 domain of *ABI3*, *FUS3*, *LEC2* and *VAL1*; add *EcoRV* and *SacI* sites at 5' end and 3' end of each B3 domain sequences.	*ABI3-B3*	F	GGCGATATCCAGGGATGGAAACCAGAAAA
		R	GTAGAGCTCGTTGTCTTACTTTAACCCCTC
	FUS3-B3	F	GGCGATATCGCACGTAAAATTGACCCAAG
		R	GTAGAGCTCGTTCCGATGCTTTTCTTGCTT
	LEC2-B3	F	GGCGATATCCAGTCCACATTTGATAACAAG
		R	GTAGAGCTCGTTCATTTTGTTTTCCCGAAT
	VAL1-B3	F	GGCGATATCCAGATCTCTGGAAATTTGAAT
		R	GTAGAGCTCGATTAGCTGCCTTCCTGGAAC
Recovery VP1::B3 border restriction sites to original sequence by mutagenesis PCR.	*VP1::ABI3-B3*	F	CTCGCAGCGGCTTCAGATAAGCGGCAGGGATGGAAACCAGAA
		F	GGGGTTAAAGTAAGACAACCGCCGGCGCAAGAGCAAGGCAGT
	VP1::FUS3-B3	F	CTCGCAGCGGCTTCAGATAAGCGGGCACGTAAAATTGACCC
		F	GCAAGAAAAGCATCGGAACCGCCGGCGCAAGAGCAAGGCAG
	VP1::LEC2-B3	F	CTCGCAGCGGCTTCAGATAAGCGGCAGTCCACATTTGATAAC
		F	TCGGGAAAACAAAATGAACCGCCGGCGCAAGAGCAAGGCAGT
	VP1::VAL1-B3	F	CTCGCAGCGGCTTCAGATAAGCGGCAGATCTCTGGAAATTTG
		F	TCCAGGAAGGCAGCTAATCCGCCGGCGCAAGAGCAAGGCAGT
	VP1::ABI3-B3 K15R	F	CGGTTTCTCTTGCAGAGGGTCTTGAAGCAAAGC
	VP1::FUS3-B3 K15R	F	CTAAGATTCCTCTTCCAAAGGGAACTCAAGAACAG
	VP1::LEC2-B3 K15R	F	CTTAGGGTTTTGTGTGAGAGGGAATTGAAGAACAG
	VP1::VAL1-B3 K16R	F	
RT-PCR and Q-PCR primers.	*CRC*	F	CCTTGCCCATCTTGGAGTATG
		R	GTTGTCGTTGACCACTTGGATC
	LEC1	F	GGTGACAAGAACAATGGTATCG
		R	GTTTCTTTGGCGTCGTCAGAG
	NOS terminator	F	GATCGTTCAAACATTTGGCA
		R	CCCATCTCATAAATAACGTC
	TUB2	F	GTTGTTCGTAAGGAAGCTGAGA
		R	GAGAAGGTAAGCATCATGCGATC

Supplementary Table S3. Primers and probes used in binding assays and ChIP-qPCR

Name	Sequences: 5'>3' (BamHI: GGATCC; EcoRI: GAATTC)
B3-BamHI for cloning	CTTGGATCCGACATTCACCACCGCCTC
B3-EcoRI for cloning	ACGGAATTCCGCTGGAACCACTGCCTTG
Sph2A-5-Biotin-probe-F	CACGGATCATGCATGGACGACACGGATCATGCATGGACGACACG
Sph2A-5-Biotin-probe-R	CGTGTCGTCCATGCATGATCCGTGTCGTCCATGCATGATCCGTG
CRC-CHIP-qPCR-F	GGAAATGAACTTTGGGCTCACG
CRC-CHIP-qPCR-R	GCTGCGACTTGTCTCGACAAC

Supplementary Table S4

***In vivo* B3 domain activities measured in transgenic Arabidopsis**

($\log_{10}$ CRC mRNA copies ng^{-1} total RNA)

	no ABA	5 µM ABA
**LEC2 B3**	1.48 +/- 0.44	4.03 +/- 0.20
**LEC2 B3 P**	0.77 +/- 0.17	3.14 +/- 1.00
**LEC2 B3 RRP**	-0.08 +/- -0.02	2.76 +/- 0.35
**ABI3 B3 KKS**	1.07 +/- 0.42	4.64 +/- 0.24
**ABI3 B3 S**	-0.22 +/- 0.42	2.93 +/- 0.37
**ABI3 B3**	-1.18 +/- 0.18	4.54 +/- 0.14

Supplementary Table S5

T-test p-values* for pair-wise comparisons of *CRC* activation *in planta*

	ABA	LEC2 B3 P	LEC2 B3 RRP	ABI3 B3 KKS	ABI3 B3	ABI3 B3 S
**LEC2 B3**	_	1.20E-01	1.88E-03	2.47E-01	1.49E-06	3.31E-03
**LEC2 B3 P**	_		4.19E-02	3.03E-01	5.49E-05	4.69E-02
**LEC2 B3 RRP**	_		.	1.20E-02	7.69E-04	3.89E-01
**ABI3 B3 KKS**	_	..	..	.	1.17E-05	1.57E-02
**ABI3 B3**	_	..	..	..	..	1.95E-02
**LEC2 B3**	+	1.07E-02	1.41E-03	2.78E-02	1.90E-02	5.60E-03
**LEC2 B3 P**	+	..	2.97E-01	2.20E-04	3.67E-05	3.41E-01
**LEC2 B3 RRP**	+	..	..	2.23E-05	2.65E-06	3.64E-01
**ABI3 B3 KKS**	+	..	..	..	3.58E-01	1.21E-04
**ABI3 B3**	+	..	..	..	..	9.26E-04

* Differences significant at $p<0.05$ *are colored red.*

Supplementary Table S6 Rate constants for biphasic dissociation of B3-DNA complex

	**b**	$\mathbf{k_{fast}}$ (s^{-1})	$\mathbf{k_{slow}}$ (s^{-1})
**ABI3-B3**	0.160 (0.005)[1]	6.02E-03 (2.96E-04)	7.72E-05 (1.49E-06)
**LEC2-B3**	0.351 (0.016)	3.58E-03 (7.94E-04)	2.18E-04 (1.05E-06)
**ABI3-B3 KKS**	0.301 (0.043)	4.14E-03 (6.09E-04)	1.14E-04 (1.12E-05)
**LEC2-B3 RRP**	0.176 (0.012)	1.55E-02 (3.53E-03)	9.68E-05 (3.18E-05)
**FUS3-B3**	0.217 (0.008)	2.17E-03 (2.98E-05)	1.22E-04 (1.40E-05)

1) Values in parentheses are standard errors for estimates from three protein concentrations.

Supplementary Table S7 On rate constants for low- and high-affinity B3-DNA binding sites

	$\mathbf{k_{on1}}$ (M^{-1} s^{-1})	$\mathbf{k_{on2}}$ ($M^{-1}s^{-1}$)
**ABI3-B3**	2.95E+03 (9.7)[1]	3.00E+03 (22.7)
**LEC2-B3**	12.56E+03 (90.6)	8.79E+03 (35.4)
**ABI3-B3 KKS**	8.02E+03 (81.0)	4.03E+03 (37.7)
**LEC2-B3 RRP**	17.21E+03 (73.9)	5.20E+03 (14.7)
**FUS3-B3**	8.25E+03 (105.7)	29.34E+03 (53.7)

1) Values in parentheses are model standard errors from three protein concentrations
